# Handling missing rows in multi-omics data integration: multiple imputation in multiple factor analysis framework

**DOI:** 10.1186/s12859-016-1273-5

**Published:** 2016-10-03

**Authors:** Valentin Voillet, Philippe Besse, Laurence Liaubet, Magali San Cristobal, Ignacio González

**Affiliations:** 1 Université de Toulouse, INRA, INPT, INP-ENVT, UMR1388, GenPhySE, Castanet-Tolosan, F-31326 France; 2 Université de Toulouse INSA, UMR5219 Institut de Mathématiques, Toulouse, F-31077 France; 3 INRAUR875 Mathématiques et Informatiques Appliquées, F-31326 Castanet-Tolosan, France

**Keywords:** Multiple omics data integration, Multivariate factor analysis, Missing individuals, Multiple imputation, Hot-deck imputation

## Abstract

**Background:**

In omics data integration studies, it is common, for a variety of reasons, for some individuals to not be present in all data tables. Missing row values are challenging to deal with because most statistical methods cannot be directly applied to incomplete datasets. To overcome this issue, we propose a multiple imputation (MI) approach in a multivariate framework. In this study, we focus on multiple factor analysis (MFA) as a tool to compare and integrate multiple layers of information. MI involves filling the missing rows with plausible values, resulting in *M* completed datasets. MFA is then applied to each completed dataset to produce *M* different configurations (the matrices of coordinates of individuals). Finally, the *M* configurations are combined to yield a single consensus solution.

**Results:**

We assessed the performance of our method, named MI-MFA, on two real omics datasets. Incomplete artificial datasets with different patterns of missingness were created from these data. The MI-MFA results were compared with two other approaches i.e., regularized iterative MFA (RI-MFA) and mean variable imputation (MVI-MFA). For each configuration resulting from these three strategies, the suitability of the solution was determined against the true MFA configuration obtained from the original data and a comprehensive graphical comparison showing how the MI-, RI- or MVI-MFA configurations diverge from the true configuration was produced. Two approaches i.e., confidence ellipses and convex hulls, to visualize and assess the uncertainty due to missing values were also described. We showed how the areas of ellipses and convex hulls increased with the number of missing individuals. A free and easy-to-use code was proposed to implement the MI-MFA method in the R statistical environment.

**Conclusions:**

We believe that MI-MFA provides a useful and attractive method for estimating the coordinates of individuals on the first MFA components despite missing rows. MI-MFA configurations were close to the true configuration even when many individuals were missing in several data tables. This method takes into account the uncertainty of MI-MFA configurations induced by the missing rows, thereby allowing the reliability of the results to be evaluated.

**Electronic supplementary material:**

The online version of this article (doi:10.1186/s12859-016-1273-5) contains supplementary material, which is available to authorized users.

## Background

**Fig. 1:**
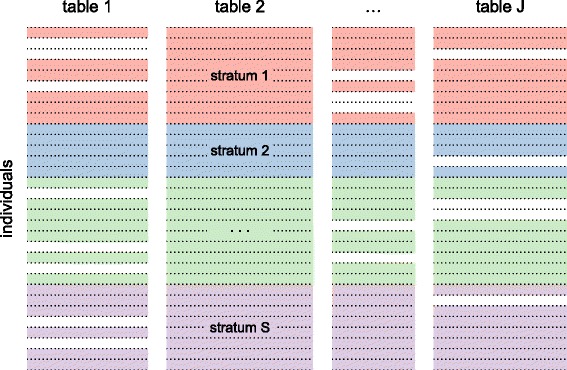
Pattern of missingness. Pattern of missing values specific to multiple data tables with missing rows for some tables of variables. The colored parts represent strata of the observed data, whereas the empty parts indicate missing data

Due to the increase in available data information [1], integrating large amounts of heterogeneous data is currently one of the major challenges in systems biology. Biological data integration provides scientists with a deeper insight into complex biological processes. However, when dealing with multiple data tables, the presence of missing values is a common situation for a variety of reasons. In omics data integration studies, it is common for some individuals to not be present in all data tables, resulting in a specific missing data pattern for multiple tables, as shown in Fig. 1. For instance, in clinical studies, this can occur when a patient forgets to fill out a form. It also can be attributable to the study design if individual data are expensive or difficult to measure.

Missing row values for a table of variables are challenging to handle because most statistical methods cannot be directly applied to incomplete datasets. In the multiple multivariate framework, several approaches have already been proposed to deal with missing row values [2]. The only methods widely available for analyzing incomplete data focus on removal of the missing rows, either by ignoring subjects with incomplete information or by replacing the missing items with plausible values (e.g., means of the observed cases). In multivariate statistical analysis, case deletion procedures can be very inefficient, discarding an unacceptably high proportion of subjects because even if the per-table rates of missing rows are low, only a few subjects may have complete data for all tables. In addition, case-deletion procedures may bias the results if the remaining subjects providing the complete data are unrepresentative of the entire sample [3, 4]. On the other hand, simple mean substitution can seriously distort the marginal and joint distribution of the variables [5] and be an issue because many statistical methods rely on estimation of the variance-covariance matrix.

Recently, two approaches have been proposed to deal with missing row values in multiple multivariate analysis. The first method, introduced by Van de Velden and Bijmolt (2006) [6], was developed in the context of generalized canonical correlation analysis. Its application in the omics framework is often limited by the size, noise and multicollinearity of the data [7, 8]. The second method, described in Husson and Josse (2013) [9], was developed in the context of multiple factor analysis (MFA). This method, designated regularized iterative MFA (RI-MFA), was derived from a method available in principal component analysis (PCA) and consists of alternating the estimation of axes and components, and the estimation of missing values [10, 11]. Here we consider an alternative method, involving a multiple imputation approach adapted to the MFA framework, and called MI-MFA.

Multiple imputation (MI) was proposed by Rubin (1987) [3] in order to estimate both the parameters of interest and their variability in a data missingness framework. It relies on the principle that a single value cannot reflect the uncertainty of the estimation of a missing value. First, MI is used to generate plausible synthetic data values, called imputations, for missing values in the data. This step results in a number (*M*) of imputed datasets in which the missing data are replaced by random draws of plausible values according to a specific statistical model. The second step consists of analyzing each imputed dataset using a statistical method that estimates the parameters of interest. This step results in *M* analyses (instead of just one) which differ only because the imputations differ. Finally, MI combines all the results together to obtain a single consensus estimate, thereby combining variation within and across the *M* imputed datasets. Under fairly liberal conditions, this last step results in statistically valid estimates that properly reflect sampling variability.

The major challenge in MI involves generating possible values for each missing observation. Statistically advanced imputation procedures can therefore be used for this. Two general approaches are often used for imputing multivariate data: joint modeling (JM) [12] and fully conditional specification (FCS), also known as multivariate imputation by chained equations (MICE) [13, 14]. JM involves specifying a multivariate distribution for the missing data and drawing imputations from their conditional distributions by Markov chain Monte Carlo (MCMC) techniques. FCS specifies the multivariate imputation model on a variable-by-variable basis using a set of conditional models, one for each incomplete variable.

The key issue in JM is appropriate specification of the multivariate distribution. A multivariate normal model has often been used as it is computationally tractable (because only the mean vector and the variance-covariance matrix need to be estimated). This model has even been used when some of the variables are not Gaussian. However, the main weakness of JM is that it can only be applied when the imputation involves a small number of variables. This is not very common in omics datasets that are often composed of tens of thousands of variables, or more. FCS allows greater flexibility than JM in creating multivariate models. Indeed, FCS can use specialized imputation models by separately defining the conditional densities for each variable, even if this can require a considerable amount of work. When the number of variables is large, it is often impractical and too computer-intensive to test and develop the best models for each variable. As an alternative to the JM and FCS approaches, we propose using the hot-deck imputation approach [5]. This approach is a nonparametric imputation method that resolves the most important limitation of the JM and FCS approaches as it can be applied to data tables containing more than just a few variables.

When using the MI method, special attention must be given to the process that gave rise to the missing data, referred to as the missing data mechanism. Most methods for generating multiple imputations, fully-, semi- and non-parametric methods, assume that the mechanism responsible for missing data is ignorable [5, 15]. Briefly, if the missing data mechanism is ignorable, then the analysis can focus on the observed values rather that also having to model the process that resulted in certain values being observed and certain values being missing. If the assumption of an ignorable missing-data mechanism is valid, then statistical methods that rely on that assumption can be expected to produce results with minimal bias. One way that the missing-data mechanism can be viewed as ignorable is if the missing data are missing completely at random (MCAR). For data to be MCAR, there must not be any systematic differences between the cases that have missing items and the cases that are fully observed. In microarray experiments, technical failure, low signal-to-noise ratio and measurement errors can for instance be considered as sources of MCAR patterns. The missing-data mechanism can also be viewed as ignorable under the less restrictive missing at random (MAR) scenario, which allows missingness to depend on observed variables but not on unobserved variables. Late-stage cancer patients, as compared to early-stage cancer patients, unfortunately have more chance of dropping out of follow-up studies, which may result in a MAR pattern in a clinical data table. When the ignorability assumption does not hold, the imputation needs to be drawn from the posterior distribution of the missing data given the complete data and the missingness mechanism. Non-ignorable missing data occurs frequently in mass-spectrometry-based experiments. Measures too close to the limit of detection of the instrument are censored, resulting in a higher rate of missing values. The probability of being missing is, in this particular case, directly dependent on the intensity value. In this paper, we decided to focus on models with ignorable missing-data mechanisms.

## Methods

### Mathematical basis of multiple factor analysis (MFA)

MFA [16] is devoted to the simultaneous exploration of multiple data tables where the same individuals are described by several tables of variables. In MFA, the number and the type of variables (quantitative or categorical) may vary from one table to another, but within each table the nature of the variables is the same. Here we focus on quantitative variables. The aims of MFA are similar to those of PCA, namely to study the similarities between individuals from a multidimensional point of view, to analyze the relationships between variables and characterize individuals based on these relationships. However, beyond these conventional uses, MFA can also be used to study the links between tables of variables and to compare the information contributed by each table.

MFA analyzes a set of *J* data tables ***K***_1_,…,***K***_*J*_, where each ***K***_*j*_ corresponds to a table of quantitative variables measured on the same *I* individuals (for a schematic overview of MFA see Additional file 1: Figure S1). The core of MFA is a PCA in which weights are assigned to variables. More formally, the matrix of variance-covariance associated with each data table ***K***_*j*_ is decomposed by PCA and its largest eigenvalue ${\lambda _{1}^{j}}$ is derived. Then, each variable belonging to ***K***_*j*_ is weighted by $1/\sqrt {{\lambda _{1}^{j}}}$. Finally, a global PCA is performed on the merged and weighted data table ***K***=[***K***_1_,…,***K***_*J*_] to obtain the configuration ***F*** (the scores matrix or principal components). The main reason for the weighting step is to remove from each table all information related to its own dimensionality or variance. Therefore, no single table can dominate the first dimension of the global analysis.

MFA provides the same graphical representations as PCA (i.e., representation of individuals and variables) but also, due to the table structure, specific representations such as the table representation and the superimposed representation are available [16].

### The multiple imputation multiple factor analysis approach (MI-MFA)

To deal with multiple tables with missing rows, we propose the MI-MFA approach, a multiple imputation (MI) adapted to the framework of MFA. The aim of our method is not to get the best possible estimations of the missing values, but to replace them with plausible values in order to provide estimates of the MFA configurations. According to MI methodology, the MI-MFA approach is carried out by performing the following three steps:

- 1. Imputation: generate *M* different imputed datasets ***K***^(1)^,…,***K***^(*m*)^,…,***K***^(*M*)^ of ***K***.
- 2. MFA analysis: perform an MFA on each ***K***^(*m*)^ imputed dataset leading to *M* different configurations ***F***_1_,…,***F***_*m*_,…,***F***_*M*_.
- 3. Combination: find a consensus configuration between all ***F***_1_,…,***F***_*M*_ configurations.

**Fig. 2:**
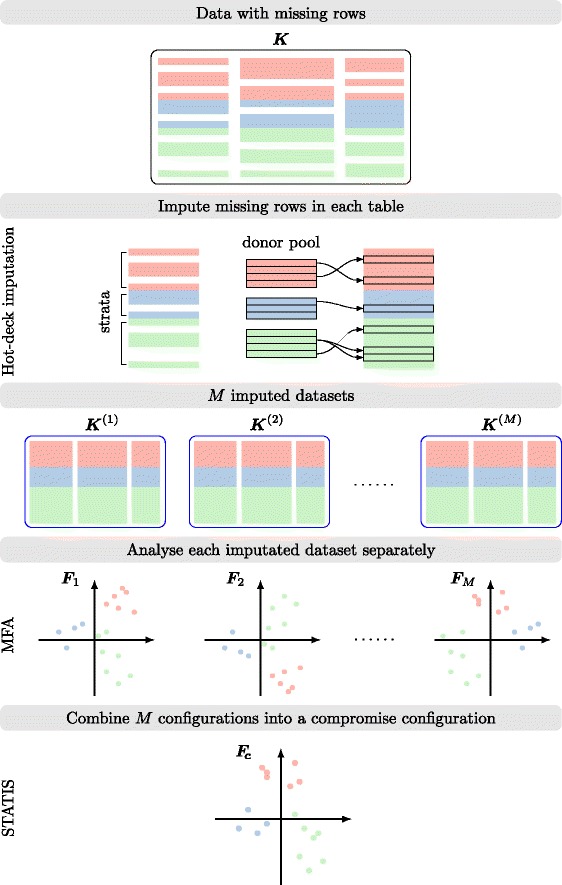
Overview of our MI-MFA approach to handling missing rows in multi-omics data integration. The *top* part of the graphic indicates that analysis starts with observed, incomplete data tables ***K***. In a second step, multiple imputation is performed using the hot-deck imputation approach: *M* imputed versions ***K*** ^(1)^,…,***K*** ^(*M*)^ of ***K*** are obtained by replacing the missing values by plausible data values. These plausible values are drawn from donor pools. The imputed sets are identical for the non-missing data entries, but differ in the imputed values. The third step is to estimate the configuration matrix ***F***_*m*_ for each imputed dataset ***K*** ^(*m*)^ using MFA. The estimated configurations differ from each other because their input data differ. The last step is to combine the *M* estimated configurations ***F***_1_,…,***F***_*M*_ into a compromise configuration ***F***_***c***_ using the STATIS method

These steps are outlined in Fig. 2 and described in detail in the following sections.

#### Generating imputed data: multiple hot-deck imputation

Hot-deck imputation involves replacing missing values of one or more variables with available values from a similar unit [17]. The observation from which these available values are taken for imputation is called the donor and the observation with the missing value, which receives the donor’s value, is the recipient. The donor can be randomly selected from a set of potential donors, called the donor pool. Selection of a suitable donor pool is not an easy task and is beyond the scope of this article [18, 19]. The general principle is to choose donor units that are as close as possible to the recipient with respect to some *affinity score*. Affinity is defined in terms of the degree to which each potential donor matches the recipient’s values across all variables other than the one being imputed. Intuitively, in the framework of stratified multiple omics tables, the donor pool can be formed of available individuals belonging to the same stratum (e.g. cancerous cell line, treatment, etc.) and the same omics table as the recipient.

Multiple hot decking differs from other forms of hot decking by using several donors for a single recipient [20]. Multiple hot-deck imputation proceeds as follows. Let ***K***=[***K***_1_,…,***K***_*J*_] be the merged data table containing missing rows with strata *s*=1,…,*S*, then carry out the following steps (see Fig. 2, hot-deck imputation step):

**Step 1.** Create donor pools by taking donors belonging to the same stratum *s* and the same table ***K***_*j*_ as the recipient. Recipients within the same stratum have the same donor pool. Suppose always that there is a large enough number of donors for recipients in each stratum.

**Step 2.** For each recipient in ***K***_*j*_, impute the missing individual by drawing randomly with replacement a donor from the corresponding donor pool. Repeat this procedure until all missing individuals in the *J* tables have been imputed.

**Step 3.** Repeat Step 2 until *M* different imputed datasets ***K***^(1)^,…,***K***^(*M*)^ of ***K*** are obtained.

By conducting the imputations in this way, it is reasonable to assume that the within-unit between-variables multivariate relationships are preserved.

#### The combination procedure: the STATIS method

The question that arises after using MI in an MFA framework is how should all the configurations resulting from the analyses be combined to obtain a single unique estimate of the *consensus* configuration? While averaging is an appropriate combination procedure in many other statistical techniques, it is not recommended for MFA due to possible reflection, dilation or rotation of the different configurations with respect to each other [21]. Here we consider an alternative approach by implementing the STATIS method which provides a compromise configuration balancing all configurations.

The STATIS method [22] (which stands for *Structuration des Tableaux à Trois Indices de la Statistique* in French) is a generalization of PCA used to simultaneously study several tables of variables collected on the same individuals. The goal of this method is to analyze the structure of the individual tables (i.e., the relation between the individual tables) and to derive from this structure an optimal set of weights for computing a common configuration of the observations. The solution obtained, called the compromise, is the configuration agrees the most with all other configurations. An overview of the STATIS method is presented in Additional file 1: Figure S2.

STATIS analyzes a set of *N* tables ***X***_1_,…,***X***_*N*_, where each ***X***_*n*_ is a table of quantitative variables measured on the same individuals. The first stage of STATIS consists of calculating a matrix of cross-products between individuals for each table $\boldsymbol {W}_{\!\!n} = \boldsymbol {X}_{\!n} \boldsymbol {X}_{\!n}^{T}$ (***A***^*T*^ means the transpose of a vector or a matrix ***A***) reflecting the similarities between individuals within this table. The use of matrices ***W***_*n*_ instead of ***X***_*n*_ simplifies the computation because it obviates the determination of rotations when matching the ***X***_*n*_. The basic idea in STATIS is then to find a compromise space $\boldsymbol {W_{\!c}} = \sum _{n=1}^{N} \alpha _{n} \boldsymbol {W}_{\!\!n}$ that globally balances these cross-product matrices by choosing a suitable optimal set of weights *α*_1_,…,*α*_*N*_. These weights are obtained from the PCA of the matrix ***R*** whose generic term ***R***_*jk*_ gives the cosine between tables (also known as the RV-coefficient [23]) defined as:

$$\boldsymbol{R}_{\!jk} = \frac{\text{trace}(\boldsymbol{W}_{\!\!j}^{T}\, \boldsymbol{W}_{\!k})}{\sqrt{\text{trace}(\boldsymbol{W}_{\!\!j}^{T}\, \boldsymbol{W}_{\!\!j})\cdot \text{trace}(\boldsymbol{W}_{\!k}^{T}\, \boldsymbol{W}_{\!k})}}\;, $$

where the trace is the sum of the main diagonal elements of a square matrix. The first eigenvector obtained from the PCA of ***R*** represents the “agreement between tables”. Its elements are normalized in such a way that their sum is equal to 1 and used as weights *α*_*n*_ in order to define ***W***_***c***_. Tables with larger values of *α*_*n*_ are more similar to the other tables and therefore will have a larger weight, while the weight of the “outlier tables” will be closer to zero with respect to the other weights. The principal components from the PCA of ***W***_***c***_ then gives the coordinates of the individuals in the compromise space, called the *compromise* configuration.

#### Implementation of MI-MFA

The MI-MFA algorithm can be summarized as follows (see Fig. 2):

**Step 0.** Start with an observed, incomplete dataset ***K***. Define the number of imputations *M* and the dimensionality *d* of the compromise configuration.

**Step 1.** Perform multiple hot-deck imputation. For *m*=1,…,*M*:

- Obtain an imputed version ***K***^(*m*)^ of ***K***, such that, $\boldsymbol {K}^{(m)}\neq \boldsymbol {K}^{(m')}\phantom {\dot {i}\!}$ for *m*≠*m*^′^. The imputed datasets are identical for the non-missing data entries, but differ for the imputed values. The imputed version of the data is obtained by using the hot-deck imputation approach.
- Perform an MFA using *d* components on the imputed dataset ***K***^(*m*)^ to obtain the configuration ***F***_*m*_.

**Step 2.** Perform a STATIS on the set of configurations ***F***_1_,…,***F***_*M*_ to obtain ***F***_***c***_, the compromise configuration.

Note that the number of dimensions *d* used in the algorithm has to be chosen a priori. However, the number of dimensions does not affect the estimation of the imputed values and the estimation of the compromise configuration. Moreover, for given ***K***^(1)^,…,***K***^(*M*)^ imputed datasets, solutions provided by the algorithms are nested (the solution with *d* dimensions is included in the solution with *d*+1 dimensions). Since the core of MI-MFA is a weighted PCA, the strategies suggested to choose the number of components in PCA can be adapted to MI-MFA, but work needs to be done to validate the quality of these extensions.

### How many imputations?

When using MI, one of the uncertainties concerns the number *M* of imputed datasets needed to obtain satisfactory results. The number of imputed datasets in MI depends to a large extent on the proportion of missing data. The greater the missingness, the larger the number of imputations needed to obtain stable results. However, in multiple hot-deck imputation, the number of imputed datasets is limited by the size of the donor pools. In any case, the total number of possible imputations *M*_*total*_ can be calculated before applying the imputation approach (see Additional file 2). If *M*_*total*_ is small (*M*_*total*_≤50), then *M*=*M*_*total*_ can be used in MI-MFA. The appropriate number of imputations can be informally determined by carrying out MI-MFA on *N* replicate sets of *M*_*l*_ imputations for *l*=0,1,2,…, with *M*_0_<*M*_1_<*M*_2_<⋯<*M*_*total*_, until the estimate compromise configurations are stabilized. More precisely, this approach can be carried out by applying the following steps:

**Step 0.** Start with an observed, incomplete dataset ***K***. Define the number of imputations *M*_*l*_ with *M*_0_<*M*_1_<*M*_2_<⋯<*M*_*total*_ and the number *N* of replicate sets of *M*_*l*_ imputations.

**Step 1.** Create collections $\mathcal {I}_{n}^{M_{l}}$, *n*=1,…,*N*, each one containing *M*_*l*_ different imputed datasets of ***K***, such that, $\mathcal {I}_{n}^{M_{l}}\neq \mathcal {I}_{n'}^{M_{l}}$, for *n*≠*n*^′^ and $\mathcal {I}_{n}^{M_{l-1}}\subset \mathcal {I}_{n}^{M_{l}}$ for *M*_0_<*M*_1_<*M*_2_<⋯<*M*_*total*_.

**Step 2.** For *n*=1,…,*N*, perform an MI-MFA using $\mathcal {I}_{n}^{M_{0}}$, to obtain *N* different compromise configurations $\boldsymbol {F_{\!c}}_{1}^{\!M_{0}},\dots,\boldsymbol {F_{\!c}}_{N}^{\!M_{0}}$.

**Step 3.** Let *l*=1.

For *n*=1,…,*N*,

- perform an MI-MFA using the collection $\mathcal {I}_{n}^{M_{l}}$, to obtain a compromise configuration $\boldsymbol {F_{\!c}}_{n}^{\!M_{l}}$;
- calculate $r_{n}^{\,l}=r(\boldsymbol {F_{\!c}}_{n}^{\!M_{l}},\,\boldsymbol {F_{\!c}}_{n}^{\!M_{l-1}})$, a measure of the distance or correlation between configurations (for example the RV coefficient [23]).

**Step 4.** Calculate $\overline {r}^{\,l}=\frac {1}{N}\sum _{n=1}^{N} r_{n}^{\,l}$ (or $\sigma (\overline {r}^{\,l})$ the standard error of $\overline {r}^{\,l}$).

**Step 5.** Repeat steps 3 to 4 for *l*=2,3,… until the differences between two subsequent $\overline {r}^{\,l}$ (or $\sigma (\overline {r}^{\,l})$) become smaller than a certain convergence criterion.

### Uncertainty of MI-MFA solutions

In an MI-MFA framework, after estimating the configurations from the imputed datasets, a new source of variability due to missing values can be taken into account. Here we describe two approaches to visualize the uncertainty of the estimated MFA configurations attributable to missing row values. First, an individual plot for all estimated MFA configurations is constructed. The individual plot is obtained by projecting each estimated MFA configuration onto the compromise configuration (named the trajectories by Lavit [22]). Each individual is represented by *M* points, each corresponding to one of the *M* MFA configurations. Confidence ellipses and convex hulls can then be constructed for the *M* configurations for each individual. The computed convex hull results in a polygon containing all *M* solutions. All individuals have confidence areas, even those without missing values. Indeed, even if only the estimation of missing values is the only change, this will have a possible impact on all MFA parameters. Therefore, the area of such an ellipse (or convex hull) provides an insight into the uncertainty of the estimated configuration. The larger the area of an ellipse (convex hull), the more uncertain the exact location of the individual. Thus, when the area of an ellipse is large, the scientist should remain really careful regarding its interpretation.

### Performance of the method

We conducted two case studies to assess the performance of our method. Instead of using theoretical distributions to generate simulated data, our studies were based on two real datasets, denoted as the original datasets. Subsequently, specific patterns of missingness were created in these datasets as illustrated in Fig. 1, resulting in what we called the incomplete datasets. This approach was used in order to more closely mirror situations that may occur in the omics context. Next, missing row values were estimated and the resulting complete datasets were referred to the imputed datasets.

We then compared our MI-MFA method to the RI-MFA approach [9] and the mean variable imputation MFA (MVI-MFA) method, in which the missing values are simply replaced by the mean of each variable after which an MFA is performed on the imputed dataset. This latter approach was considered as the common base for comparing the MI-MFA and RI-MFA methods. For each configuration obtained using MI-, RI- and MVI-MFA, the similarity between the configuration solution and the true configuration (based on an MFA using the original dataset) was assessed from the RV coefficient [23]. The RV coefficient, which ranges from zero to one, can be interpreted as a correlation coefficient between two matrices, which allows the relative positions of objects to be compared from one configuration to another.

We also provide comprehensive graphical comparisons of the true vs. the MI-, RI- or MVI- MFA configurations. The individuals from both configurations are drawn in a same plot and connected by an arrow, the length of which indicates the divergence between the two configurations.

### Implementation of the analyses

All analyses were performed using the R computing environment [24]. MFA was performed using the *MFA* function of the *FactoMineR* R package [25]. The *statis* function of the *ade4* R package [26] was used to determine the compromise configuration. The RI-MFA method is implemented in the *imputeMFA* function available in the *missMDA* R package [27]. Note that the number of components *ncp* used to predict the missing entries in the *imputeMFA* function has to be chosen a priori. This choice is crucial and difficult [9]. As the true configuration was known in our case, the number of components *ncp* was chosen to minimize the RV coefficient between the true and the *imputeMFA* configurations.

The appropriateness of the results from MI-, RI- and MVI- MFA was then determined by comparing the configurations resulting from these three strategies with the true MFA configuration. Due to a possible lack of alignment (order change, sign reversal of the components and rotation) between two configurations (the true vs. the MI-, RI- or MVI- MFA configuration), it was necessary to align them before being compared. Ordinary Procrustes Analysis [28] was used to align these configurations prior to their comparison.

### Datasets

#### Liver toxicity

The datasets originated from a liver toxicity study [29] in which 64 male rats of the inbred Fisher F344/N strain were exposed to toxic doses of acetaminophen (paracetamol) in a controlled experiment. Necropsies were performed 6, 18, 24 and 48 h after exposure and mRNA was extracted. The data consisted of the expression of 3,116 genes and 10 clinical variables considered to be markers of liver injury. The 64 subjects (rats) were cross-classified in eight strata (or treatments) according to two factors:

- exposure time: 6, 18, 24 and 48 h;
- toxic doses of acetaminophen: high (1500 mg/kg or 2000 mg/kg) or low (50 mg/kg or 150 mg/kg).

Eight subjects per stratum were included. These datasets were downloaded from the *mixOmics* R package [30].

#### NCI-60 data

The NCI-60 dataset contained transcriptomic [31] and proteomic [32] tables for a collection of 60 cell lines from the National Cancer Institute (NCI-60). The NCI-60 panel included cell lines derived from various cancer types: colon (7 cell lines), renal (8), ovarian (6), breast (8), prostate (2), lung (9) and central nervous system (6), as well as leukemia (6) and melanoma (8). The gene expression profiles used here were generated using an Agilent platform [31] and downloaded from Cellminer [33]. Data were log_2_-transformed. To facilitate data interpretation and computation, the transcriptomic data were filtered to exclude probes that did not map to an official HUGO gene symbol and to retain only the probe with the highest average value when multiple probes mapped to the same gene, as previously described in [34]. Gene invariants across all 60 cell lines, corresponding to genes without any effect between cancer types, were also removed. Filtering produced a dataset of 1,433 genes. The NCI-60 proteome table was also downloaded from Cellminer [33]. Proteomic data were obtained using high-density reverse-phase lysate microarrays [32]. Data were log_2_-transformed and protein abundance levels were available for 162 proteins [32].

## Results

### Liver toxicity data analysis

**Fig. 3:**
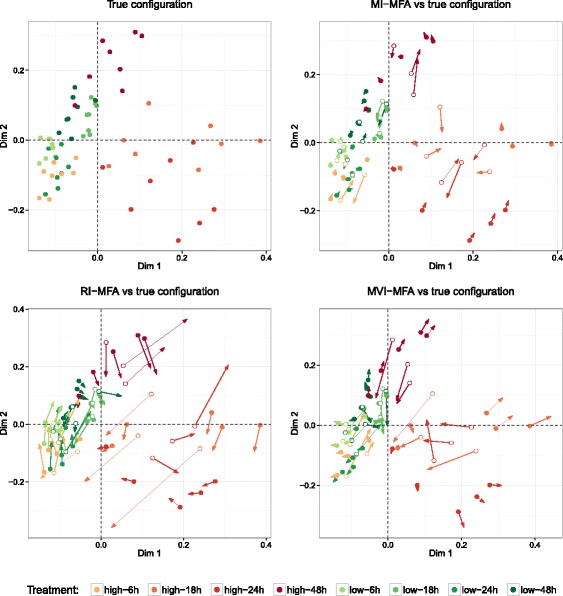
Comparison of MFA configurations for the liver toxicity data analysis. Representation of the individuals on the two-dimensional configuration obtained by performing MFA on the complete dataset (*top left*) and, MI-MFA (*top right*), RI-MFA (*bottom left*) and MVI-MFA (*bottom right*) versus the true configuration. Dots and arrows represent the projected coordinates from the true configuration and the imputation MFA method, respectively. The length of the line joining dots and arrows is proportional to the divergence between the projected coordinates. The color of each individual (*dot-arrow*) reflects the treatment (see legend). Imputed individuals from the transcriptomic dataset are represented by empty circles

A specific pattern of missing values was created as illustrated in Fig. 1. To obtain an incomplete dataset, three individuals per stratum were randomly removed from the transcriptomic table. For this specific pattern, there were 3×8=24 missing individuals. MI-MFA was then performed on the incomplete dataset using *M*=30 imputed datasets. RI-MFA, and MVI-MFA were also performed. Figure 3 shows the divergence of the MI-, RI- and MVI-MFA configurations from the true configuration. As can be seen, the configuration obtained with MI-MFA was very close to the true configuration (Fig. 3, top right). This result was confirmed by the high RV coefficient (0.96 for the first two dimensions). The configurations obtained with RI-MFA and MVI-MFA were more distorted and less close to the true configuration with RV coefficients of 0.77 and 0.84 respectively (Fig. 3, bottom).

**Fig. 4:**
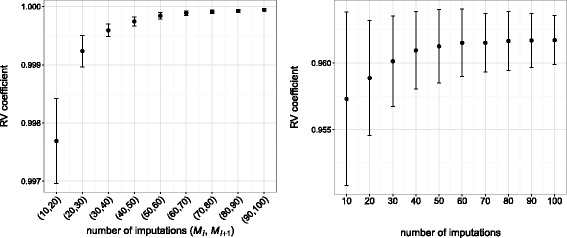
Stability of the estimated MI-MFA configurations using different numbers of imputations. MI-MFA configurations were obtained for the following numbers of imputations: *M*_*l*_=10 *l*, for *l*=1,…,10. *Left*: RV coefficient between configurations obtained by MI-MFA with *M*_*l*_ and *M*_*l*+1_ imputations. *Right*: RV coefficient between the configuration obtained by MFA on the complete dataset and MI-MFA on the incomplete dataset with *M*_*l*_ imputations. The values shown are the mean RV coefficients for the *N*=30 two-dimensional configurations as a function of the number of imputations. Error bars represent the standard deviation of the RV coefficients

The number *M* of imputed datasets in MI-MFA for the above incomplete liver toxicity data was determined as described in the *How many imputations* section. Collections of size *N*=30 were generated for each of the following numbers of imputations: *M*_*l*_=10 *l*, for *l*=1,…,10. The stability of the estimated MI-MFA configurations was then determined by calculating the RV coefficient between the configurations obtained using *M*_*l*_ and *M*_*l*+1_ imputations (see Fig. 4, left). As the true configuration was known, we also described the stability of the estimated MI-MFA configurations by calculating the RV coefficients between the true configuration and those obtained using *M*_*l*_ imputations (see Fig. 4, right). Although the missing information is substantial, Fig. 4 shows that only a slight increase in precision was obtained by using more than 30 imputations.

MI-MFA was also applied to different scenarios of missingness. First, MFA was performed on the original dataset to obtain the true configuration. Secondly, individuals were randomly removed from each stratum in the original transcriptomic table. For this, three scenarios were considered for the number of missing rows (i.e. low, medium and high), in which there were respectively one, two and three missing rows per stratum. The total number of missing rows per scenario was therefore 8, 16 and 24 respectively. Thirdly, for each missingness scenario, 50 incomplete datasets were randomly chosen for analysis.

**Fig. 5:**
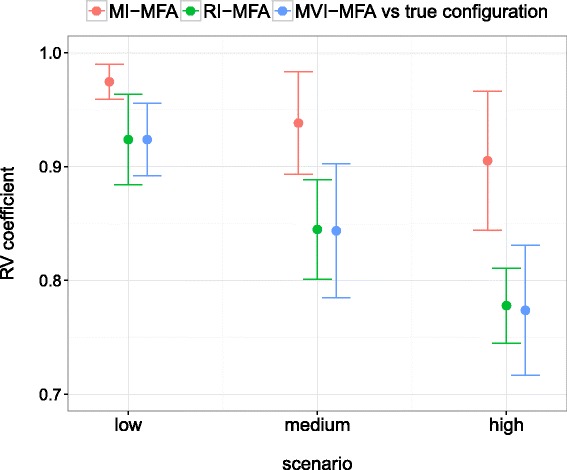
Performance on liver toxicity data according to the number of missing rows. RV coefficients between the configuration obtained by MFA on the complete dataset and either MI-MFA (*red line*), RI-MFA (*green line*) or MVI-MFA (*blue line*) on the incomplete dataset. The values shown are the mean RV coefficients for the 50 two-dimensional configurations for each missingness scenario: low, medium and high (see text for more details). Error bars represent the standard deviation of the RV coefficients

As previously, MI-MFA was performed on each incomplete dataset using *M*=30 imputed datasets. RI-MFA, as well as MVI-MFA, were also computed. The RV coefficients between the true configuration and the configurations obtained using each method (for the first two dimensions) were then calculated. Figure 5 shows the mean of the RV coefficients for the 50 two-dimensional configurations as a function of the missingness scenario for each method. Note that the average results using MI-MFA were always better than with RI-MFA or MVI-MFA, whatever the scenario. The RV coefficients between the true configuration and the MI-MFA configuration were close to one and remained satisfactory even when the number of missing row values was high, and the results obtained with the RI-MFA and MVI-MFA decreased significantly.

The performance of the MI-MFA procedure was then further investigated for more complex scenarios of missingness. More precisely, missing row values were inserted into each stratum (e.g. high-6h treatment) of the original dataset (including both transcriptomic and clinical tables) according to the scenarios illustrated in Table 1.

**Table 1:** Scenarios of missingness for the liver toxicity data analysis

Number of missing rows			
Scenario	Transcriptome	Clinical	# cases
1	1	1	56
2	2	1	168
3	1	2	168
4	3	1	280
5	2	2	420
6	3	2	560
7	4	1	280

Number of missing rows inserted in each stratum of the original dataset, including both transcriptomic and clinical data, for incomplete data creation. The # cases indicate the number of possibilities of incomplete cases per stratum

**Fig. 6:**
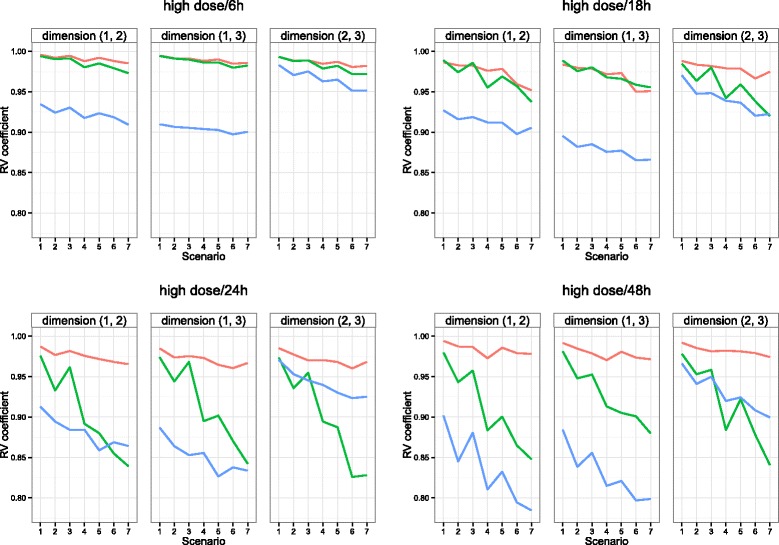
Performance on liver toxicity data with missing individuals in the high-dose treatment. Average RV coefficient between the configuration obtained by MFA on the complete dataset and either MI-MFA (*red line*), RI-MFA (*green line*) or MVI-MFA (*blue line*) on the incomplete dataset. Results are given for all of the first three two-dimensional possible configurations as a function of the scenarios presented in Table 1. The discrete RV values are joined by lines for ease of understanding. The performance of the different methods on the liver toxicity data with missing individuals in the low-dose treatment are presented in Additional file 1: Figure S3

Twenty incomplete datasets were then selected at random from each stratum (treatment) and each scenario. All analyses (MI-MFA, RI-MFA and MVI-MFA) and the calculation of the RV coefficient were performed in the same way as previously described. Figure 6 (and Additional file 1: Figure S3) shows the means of RV coefficients for the two-dimensional configurations as a function of the scenarios for each method.

For almost all the scenarios, the average results obtained with MI-MFA were better than with the other methods. As the number of missing row values increased (Fig. 6 and Additional file 1: Figure S3), the results obtained with the RI-MFA and MVI-MFA algorithms worsened rapidly, especially when the dimension increased, whereas the results with the MI-MFA approach were still satisfactory.

### NCI-60 data analysis

To confirm the performance of our method, MI-MFA was also performed on the NCI-60 dataset. A pattern of missing values was created as illustrated in Table 2. One or two individuals were removed per table for all types of cancer cell lines, except for the prostate cancer line (which only had two individuals). This specific pattern would reflect a study in which a lot of rows were missing.

**Table 2:** Scenarios of missingness for the NCI-60 data analysis

Number of missing rows			
Cell line	Transcriptome	Proteome	# cases
Breast	1	0	5
CNS	1	1	60
Colon	2	0	42
Lung	2	2	3024
Leukemia	1	1	60
Melanoma	2	2	5040
Ovarian	1	1	84
Prostate	0	0	//
Renal	2	1	504

Number of missing rows inserted in each stratum (cell line type) of the original dataset, including both transcriptomic and proteomic data, for incomplete data creation. The # cases indicate the number of possibilities of incomplete cases per stratum

**Fig. 7:**
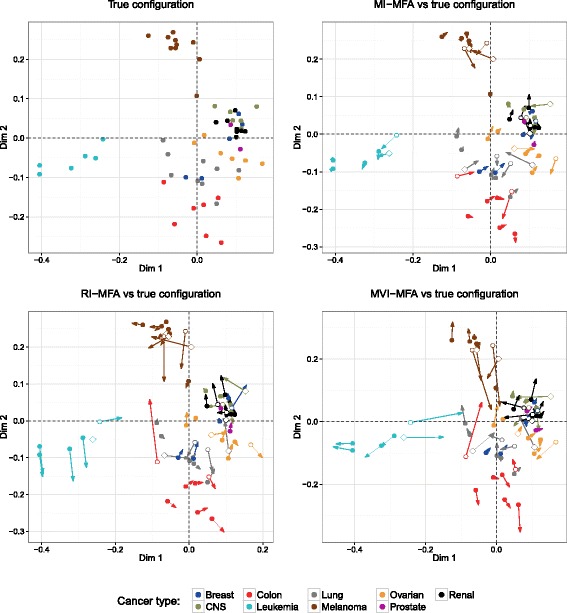
Comparison of MFA configurations for NCI-60 data analysis. Representation of the individuals on the two-dimensional configuration obtained by MFA on the complete dataset (*top left*) and, MI-MFA (*top right*), RI-MFA (*bottom left*) and MVI-MFA (*bottom right*) versus the true configuration. Dots and arrows represent the projected coordinates from the true configuration and the imputation MFA method respectively. The length of the line joining dots and arrows is proportional to the divergence between the projected coordinates. Each individual (*dot-arrow*) is colored according to the cancer type (see legend). Imputed individuals from transcriptomic and proteomic data are represented by empty circles and diamonds, respectively

To compare the MFA configurations, one incomplete dataset was chosen from a large range of possibilities of incomplete datasets (6×10^14^) according to the scenario of missingness illustrated in Table 2. We then computed our MI-MFA method on this incomplete dataset by using *M*=50 imputed datasets. As with the liver toxicity data, RI-MFA and MVI-MFA were also performed. We chose *M*=50 in order to achieve stable results. Figure 7 shows the divergence of the MI-, RI- and MVI-MFA configurations from the true configuration. For this specific example, the MI-MFA configuration was closest to the true configuration (Fig. 7, top-right) with a RV coefficient of 0.97, whereas the configurations obtained with RI-MFA and MVI-MFA were more distorted with RV coefficients of 0.94 and 0.87 respectively (Fig. 7, bottom).

**Fig. 8:**
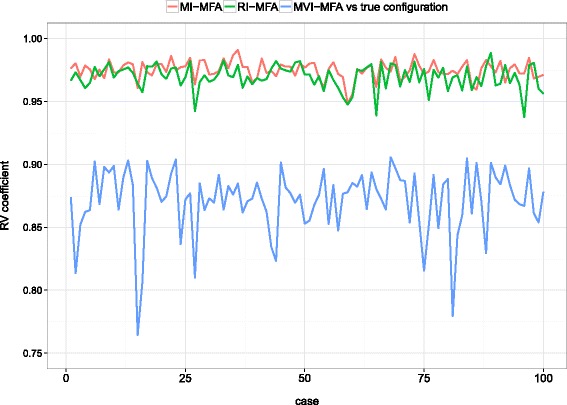
Performance on NCI-60 data. RV coefficient between the configuration obtained by MFA on the complete dataset and either MI-MFA (*red line*), RI-MFA (*green line*) or MVI-MFA (*blue line*) on the incomplete dataset. The results shown are the mean RV coefficients for the 50 two-dimensional configurations as a function of the cases according to the scenario represented in Table 2. The discrete RV values are joined by lines for ease of understanding

To broaden the scope of assessment to more than a single case, 100 possible cases of incomplete datasets were chosen at random among the 6×10^14^ possibilities according to the specific missingness scenario shown in Table 2. For each incomplete dataset, MI-MFA was performed using *M*=50 imputed datasets. RI-MFA and MVI-MFA were also computed. Figure 8 shows the RV coefficient for the two-dimensional configurations as a function of each case. The results obtained with MI-MFA and RI-MFA were similar, with RV coefficients of approx. 0.97, whereas the results obtained with MVI-MFA were much further from the true configuration. Thus, even with complex patterns of missingness, the MI-MFA approach still provided satisfactory results, as did RI-MFA in this case. However, unlike RI-MFA, MI-MFA took into account the variability of missing row values, as demonstrated in the following section.

### Why is it essential to evaluate uncertainty?

**Fig. 9:**
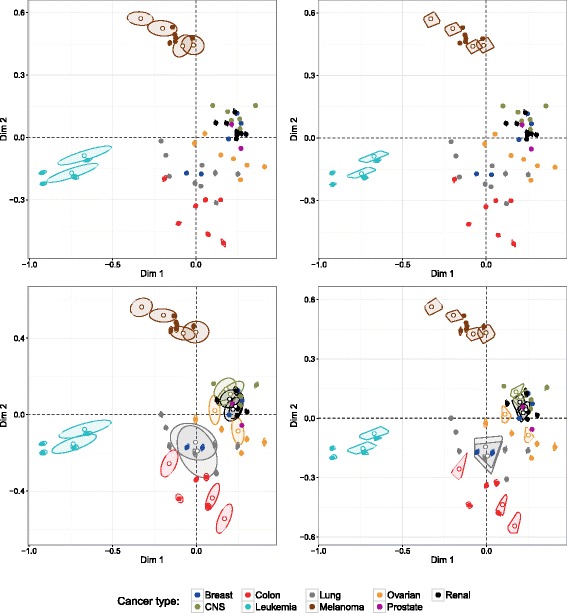
Visualization of the uncertainty induced by the missing individuals. Projection of the 50 obtained configurations on the compromise configuration from MI-MFA on the incomplete data with 10 % missing rows (*top*) and 30 % missing rows (*bottom*). The 95 % confidence ellipses (*left*) and convex hulls (*right*) show the uncertainty for each individual (*dots*). Empty circles represent imputed individuals from the transcriptomic data. For ease of understanding, not all individuals for the 50 configurations obtained were plotted

This question was addressed through an example using the NCI-60 dataset. A specific pattern of missing values was created. The missing rows were randomly introduced for four melanoma and two leukemia cancer lines in the transcriptomic table. The six inserted missing rows represented 10 % of the total number of individuals. The missing rows were inserted for specific groups of individuals that contributed substantially to the construction of the first two dimensions of the MFA on the original dataset (see Fig. 7, top left). MI-MFA was performed on the incomplete dataset using *M*=50 imputed datasets. Confidence ellipses and convex hulls were then computed from the 50 configurations projected on the compromise configuration. Figure 9 (top) shows the uncertainty due to missing rows around individuals on the compromise configuration. The use of different imputed individuals in each dataset implied slightly different configurations. Consequently, since the configurations changed, the positions of all the individuals also changed and thus all the individuals had confidence areas, even individuals not being imputed (Fig. 9, individuals represented by filled circles). However, the greatest uncertainty occurred around the imputed individuals (Fig. 9, empty circles).

In order to highlight the importance of the uncertainty of MI-MFA configurations induced by missing rows, additional rows were removed from the transcriptomic table resulting in 30 % missing rows. We then carried out MI-MFA on the incomplete dataset using *M*=50 imputed datasets. Figure 9 (bottom) shows the impact of the missing rows around individuals on the compromise configuration. As expected, the size of the ellipses (and convex hulls) of the additional missing individuals was increased. However, the size of the ellipses and convex hulls was not excessive even when 30 % of the rows were missing from the transcriptomic table.

## Discussion

In the present paper, we propose a MI approach to handle missing row values in an MFA framework and therefore resolve one of the major issues associated with multiple omics data tables. The proposed method, which we called MI-MFA, provides point estimates of MFA configurations with a notion of uncertainty due to the missing values. The aim of our method was not to obtain the best possible estimates for the missing values, but to replace them so as to be able to estimate MFA configurations.

The MI-MFA method generates *M* imputed datasets from an MFA model, where multiple hot-deck imputation is used to fill in the missing values. The hot-deck approach resolves the most important limitation of other model-based techniques (such as JM and FCS) in that it can be applied to large datasets. Furthermore, other major advantages of this method are that: (1) it is not necessary to define an explicit model for the distribution of the missing values, (2) imputations tend to be realistic since they are based on observed values, and (3) it is flexible in the sense that it can preserve complex within-unit and between-variable associations. However, a weakness is that it requires good donor-recipient matches that reflect the available covariate information. Finding such good matches is not an easy task and is beyond the scope of this article [18, 19]. In an ideal framework of stratified multiple omics data tables, donor pools would consist of the available individuals belonging to the same stratum and the same omics table as the recipient. A potential shortcoming of the method is that the donor pools might contain too few donor observations and thus introduce a risk of bias on the MFA results. Likewise, if the overall sample size is very small, then typically there are also too few potential donors. However, all imputation techniques are challenged by small sample sizes since these reduce the availability of information required to create suitable conditional statements [19].

An important aspect of our strategy is the choice of the number *M* of imputed datasets. As shown in the two case studies, we think that this number should be a good compromise between the need to obtain stable estimates and to avoid computation bottlenecks.

The STATIS method was proposed to combine the results of MI-MFA. As the core of MFA is a PCA, combining the results from MFA is the same as combining the results from PCA. Several procedures have previously been proposed to combine results from PCA such as the Mean Varimax Method (MVM) or Mean Correlation Matrix (MCM) approaches (as discussed in [35]). However, Van Ginkel and Kroonenberg [10, 35] demonstrated that Generalized Procrustes Analysis (GPA) was more suited to this purpose. GPA fits the PCA configurations obtained from the imputed datasets to a single fixed reference configuration to produce the final solution, the centroid configuration (the mean of all transformed solutions). One advantage of STATIS, as compared to MVM and MCM, is that it automatically corrects for possible reflection, dilation or rotation of the different configurations. Additionally, and contrary to the GPA procedure, the STATIS algorithm is very computer-time efficient since it is a non-iterative process. Another appealing feature of STATIS is its robust properties. As this algorithm includes weights proportional to the agreement between configurations, the results do not seem to be affected by the presence of large outliers.

Two approaches have been proposed to visualize the uncertainty of the estimated MFA configurations due to missing row values: confidence ellipses and convex hulls. These graphical representations provide scientists with considerable guidance when interpreting the significance of MFA results in a missing data framework. Indeed, ellipses and convex hull areas offer great assistance by either supporting the MFA results if they are small or suggesting that caution be exercised otherwise. It should be noted that RI-MFA (or MVI-MFA) also provides a configuration of individuals whatever the missingness pattern; however, there is no way of knowing if the results obtained are plausible and if the user can interpret the results without making any mistakes.

We have illustrated our approach by applying it to two real case studies using the liver toxicity and NCI-60 datasets. Incomplete artificial datasets with different patterns of missingness were created within these datasets. The configurations resulting from MI-, RI- and MVI-MFA were compared with the MFA configurations of the corresponding original population (the true configuration). Performance of the methods was assessed by considering the RV coefficient with respect to the true configuration.

In the liver toxicity study, the performances of the methods were compared in two different missingness settings. First the number of missing rows in each stratum of the transcriptomic table was chosen to be low (1 row), medium (2) or high (3). Secondly, seven scenarios were created by inserting missing rows in the original dataset which included both transcriptomic and clinical tables. The overall results showed that MI-MFA clearly outperformed the RI-MFA and MVI-MFA approaches in nearly all settings.

In the NCI-60 study, we illustrated the performance of our method on complex patterns of missingness where substantial numbers of rows were missing from both tables of the NCI-60 dataset. As previously, this study showed that MI-MFA clearly performed better than MVI-MFA. We also demonstrated that RI-MFA performed better than MVI-MFA. The differences between MI-MFA and RI-MFA were small, but on average slightly in favor of our method. As the purpose of this study was also to illustrate the uncertainty of MI-MFA configurations induced by missing rows, we demonstrated how the areas of the confidence ellipses and convex hulls got larger as the number of missing rows increased.

## Conclusion

We propose here a new method, MI-MFA, an extension of MFA, designed to deal with multiple tables with missing row values. MI-MFA is a useful and attractive method to estimate the coordinates of individuals for MFA configurations despite the missing rows. The study cases showed that the other proposed methods either encountered serious problems or were unable to adequately assess the accuracy due to missing data. The configurations obtained with our method were closer to the true configuration even when a significant number of individuals were missing, and thus provided better results. Moreover, the uncertainty due to the missing rows could be visualized on the compromise configuration. The software for our MI-MFA method is available in an easy-to-use code for the R statistical environment.

## Additional files

Additional file 1 Supplementary figures. Figures S1–S3. (PDF 225 kb)

Additional file 2 Calculation of the total number of possible imputations in MI-MFA. (PDF 133 kb)

Additional file 3 R code implementing the MI-MFA method. R (>=3.2) is required. (PDF 224 kb)

## Abbreviations

FCS: Fully conditional specification
JM: Joint modeling
MAR: Missing at random
MCAR: Missing completely at random
MFA: Multiple factor analysis
MI: Multiple imputation
MICE: Multivariate imputation by chained equations
MI-MFA: Multiple imputation multiple factor analysis
MVI-MFA: Mean variable imputation multiple factor analysis
PCA: Principal component analysis
RI-MFA: Regularized iterative multiple factor analysis
STATIS: Structuration des tableaux à trois indices de la statistique (in French)
