# Supplementary material for: Handling missing rows in multi-omics data integration: multiple imputation in multiple factor analysis framework
Source: BMC Bioinformatics. 2016 Oct 3;17:402. doi: 10.1186/s12859-016-1273-5 (PMC5048483; doi:10.1186/s12859-016-1273-5)
Supplement: Additional file 1 — Supplementary figures. Figures S1–S3. (PDF 225 kb) [file 12859_2016_1273_MOESM1_ESM.pdf]

Supplementary figures

Handling Missing Rows in Multi-Omics Data Integration: Multiple Imputation in Multiple Factor Analysis Framework

Valentin Voillet[1,2,3], Philippe Besse[4], Laurence Liaubet[1,2,3], Magali San Cristobal[1,2,3,4] and Ignacio González[5]

Author details
[1] INRA, UMR1388 Génétique, Physiologie et Systèmes d'Élevage, F-31326, Castanet-Tolosan, France. [2] Université de Toulouse INPT ENSAT, UMR1388 Génétique, Physiologie et Systèmes d'Élevage, F-31326, Castanet-Tolosan, France. [3] Université de Toulouse INPT ENVT, UMR1388 Génétique, Physiologie et Systèmes d'Élevage, F-31076, Toulouse, France. [4] Université de Toulouse INSA, UMR5219 Institut de Mathématiques, F-31077, Toulouse, France. [5] INRA, UMR875 Mathématiques et Informatiques Appliquées, F-31326, Castanet-Tolosan, France.

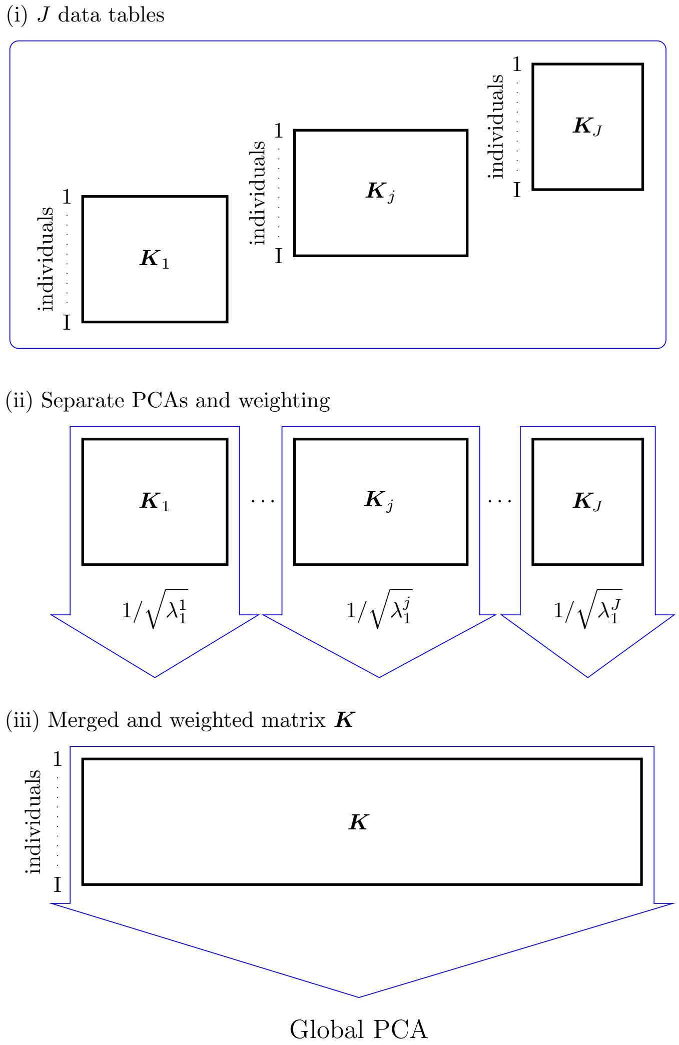

Figure S1: Schematic overview of Multiple Factor Analysis. (i) MFA analyzes a set of J-data tables, where each $\boldsymbol{K}_j$ corresponds to an omics table of quantitative variables measured on the same I individuals. (ii) J separate PCA are executed on each data table $\boldsymbol{K}_j$ and its largest eigenvalue λ_1^j is derived. Then, each variable belonging to $\boldsymbol{K}_j$ is weighted by $1/\sqrt{\lambda_1^j}$. (iii) A global analysis (PCA) is then performed on the merged and weighted data matrix $\boldsymbol{K} = [\boldsymbol{K}_1, \ldots, \boldsymbol{K}_J]$.

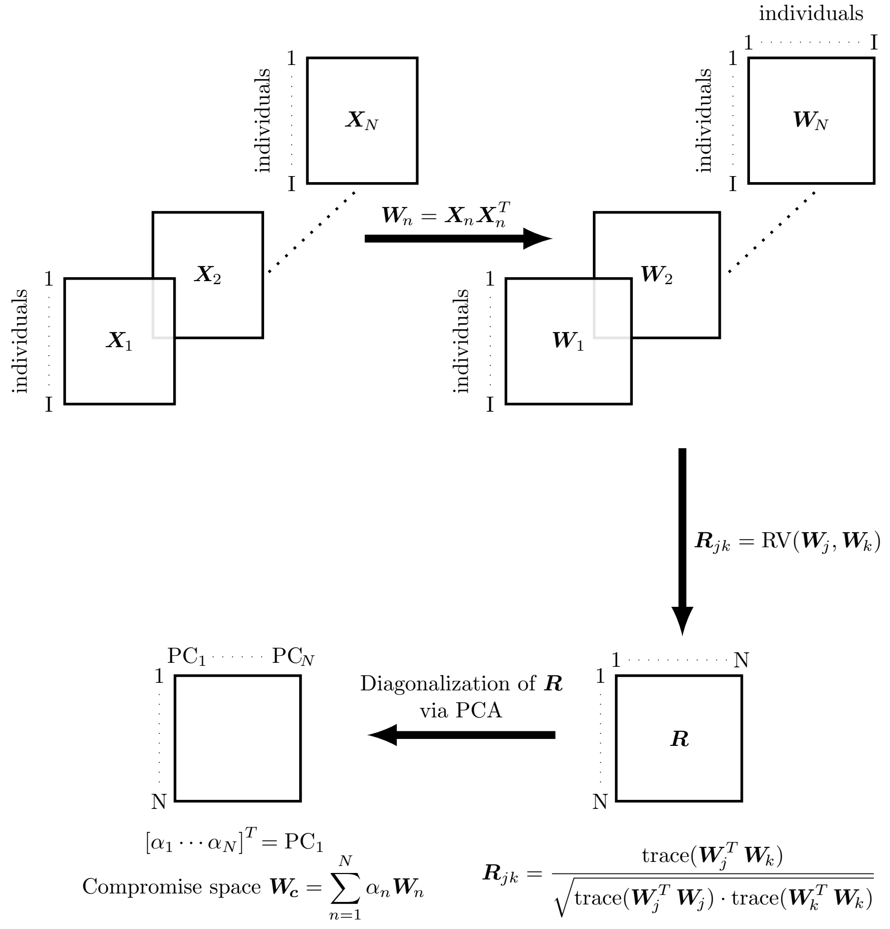

Figure S2: Schematic overview of STATIS. The top-left part of the graphic indicates that the method starts with a set of N data tables $\boldsymbol{X}_1, \ldots, \boldsymbol{X}_N$, where each $\boldsymbol{X}_n$ is a data table of quantitative variables on the same I individuals. The first stage of STATIS consists of calculating a matrix of cross-products between individuals for each table $\boldsymbol{W}_n = \boldsymbol{X}_n \boldsymbol{X}_n^T$ reflecting the similarities between individuals within this table. Second, a distance matrix $\boldsymbol{R}$ between the $\boldsymbol{W}_n$ is calculated. Third, the matrix $\boldsymbol{R}$ is diagonalized using PCA and the first eigenvector is derived. Finally, STATIS finds the compromise space $\boldsymbol{W_c} = \sum_{n=1}^{N} \alpha_n \boldsymbol{W}_n$ by a suitable optimal set of weights $\alpha_1, \ldots, \alpha_N$. These weights are obtained from coefficients of the first eigenvector (PC_1) of the diagonalization of the matrix $\boldsymbol{R}$. The PCA of $\boldsymbol{W_c}$ then gives the coordinates of the individuals in the compromise space – the *compromise* configuration.

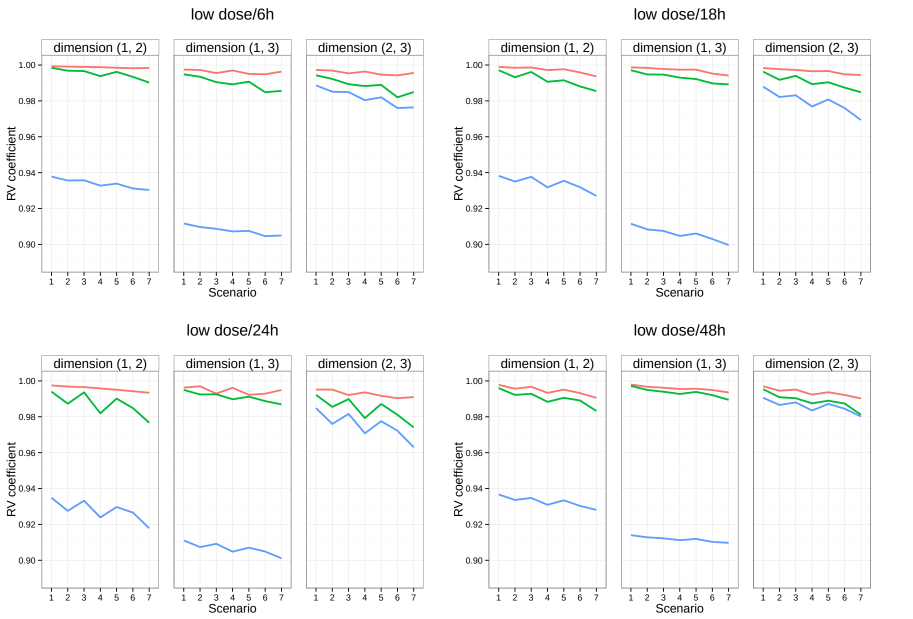

Figure S3: Performance on liver toxicity data with missing individuals in the low-dose treatment. Average RV coefficient between the configuration obtained by MFA on the complete dataset and either MI-MFA (red line), RI-MFA (green line) or MVI-MFA (blue line) on the incomplete dataset. Results are given for all of the first three two-dimensional possible configurations as a function of the scenarios presented in Table 1. The discrete RV values are joined by lines for ease of understanding.
