# Supplementary material for: Handling missing rows in multi-omics data integration: multiple imputation in multiple factor analysis framework
Source: BMC Bioinformatics. 2016 Oct 3;17:402. doi: 10.1186/s12859-016-1273-5 (PMC5048483; doi:10.1186/s12859-016-1273-5)
Supplement: Additional file 2 — Calculation of the total number of possible imputations in MI-MFA. (PDF 133 kb) [file 12859_2016_1273_MOESM2_ESM.pdf]

Calculation of the total number of possible imputations in MI-MFA

Handling Missing Rows in Multi-Omics Data Integration: Multiple Imputation in Multiple Factor Analysis Framework

Valentin Voillet [1,2,3], Philippe Besse [4], Laurence Liaubet [1,2,3], Magali San Cristobal [1,2,3,4] and Ignacio González [5]

Author details

[1] INRA, UMR1388 Gntique, Physiologie et Systmes d'levage, F-31326, Castanet-Tolosan, France. [2] Universit de Toulouse INPT ENSAT, UMR1388 Gntique, Physiologie et Systmes d'levage, F-31326, Castanet-Tolosan, France. [3] Universit de Toulouse INPT ENVT, UMR1388 Gntique, Physiologie et Systmes d'levage, F-31076, Toulouse, France. [4] Universit de Toulouse INSA, UMR5219 Institut de Mathmatiques, F-31077, Toulouse, France. [5] INRA, UMR875 Mathmatiques et Informatiques Appliques, F-31326, Castanet-Tolosan, France.

Notation

The product of a sequence of terms $x_1, x_2, \ldots, x_n$ is given by:

$$\prod_{i=1}^{n} x_i = x_1 \cdot x_2 \cdot \cdots \cdot x_n$$

The number of permutations of b elements taken from a given set of size a is defined by

$$P(a,b) = a \cdot (a-1) \cdot (a-2) \cdots (a-b+1)$$

for $a \geqslant b > 0$.

Calculation of M_{total}, the total number of possible imputations in MI-MFA

Let $\boldsymbol{K} = [\boldsymbol{K}_1, \ldots, \boldsymbol{K}_J]$ be the merged data table containing missing rows with strata $s = 1, \ldots, S$. Let obs_j^s and mis_j^s respectively be the number of observed and missing individuals in the table $\boldsymbol{K}_j$ and stratum s. The number of possible imputations for mis_j^s missing rows is then given by $P(obs_j^s, mis_j^s)$.

Thus, the total number of possible imputations M_{total} is

$$M_{total} = \prod_{j \in J^*} \left(\prod_{s \in S^*(j)} P(obs_j^s, mis_j^s) \right),$$

where J^* ($J^* \subseteq \{1, \ldots, J\}$) is the set of indexes corresponding to tables with missing rows, and $S^*(j)$ is the set of indexes of strata containing missing rows in the table $\boldsymbol{K}_j$ ($j \in J^*$).
