# Supplementary material for: Handling missing rows in multi-omics data integration: multiple imputation in multiple factor analysis framework
Source: BMC Bioinformatics. 2016 Oct 3;17:402. doi: 10.1186/s12859-016-1273-5 (PMC5048483; doi:10.1186/s12859-016-1273-5)
Supplement: Additional file 3 — R code implementing the MI-MFA method. R (>=3.2) is required. (PDF 224 kb) [file 12859_2016_1273_MOESM3_ESM.pdf]

R code implementing the MI-MFA method

Handling Missing Rows in Multi-Omics Data Integration: Multiple Imputation in Multiple Factor Analysis Framework

Valentin Voillet [1,2,3], Philippe Besse [4], Laurence Liaubet [1,2,3], Magali San Cristobal [1,2,3,4] and Ignacio González [5]

Author details

[1] INRA, UMR1388 Gntique, Physiologie et Systmes d'levage, F-31326, Castanet-Tolosan, France. [2] Universit de Toulouse INPT ENSAT, UMR1388 Gntique, Physiologie et Systmes d'levage, F-31326, Castanet-Tolosan, France. [3] Universit de Toulouse INPT ENVT, UMR1388 Gntique, Physiologie et Systmes d'levage, F-31076, Toulouse, France. [4] Universit de Toulouse INSA, UMR5219 Institut de Mathmatiques, F-31077, Toulouse, France. [5] INRA, UMR875 Mathmatiques et Informatiques Appliques, F-31326, Castanet-Tolosan, France.

Contents

1 The functions **2**
MIMFA . . . 2
plot.MIMFA . . . 6
tuneM . . . 10

2 Examples **15**

3 Session information **21**

1 The functions

MIMFA function

MIMFA	*Handling Missing Individuals in MFA*

Description

The MIMFA function estimates coordinates of individuals on the MFA components by implementing a multiple imputation (MI) approach in order to deal with multiple tables in presence of missing individuals (rows).

Usage

```
MIMFA(datasets, strata, ncomp = 2, M = NULL)
```

Arguments

datasets	a list of data tables containing the studied datasets with missing rows. Tables in the list should be arranged in samples $\times$ variables, with samples order matching in all data tables.
strata	data frame with two columns, the first one containing the individual names and the second containing the corresponding condition (stratum).
ncomp	number of MFA components. Default to 2.
M	integer, number of imputations (default `min(30, M.total)`).

Details

According to the MI methodology, missing rows are filled in by several sets of plausible values, resulting in m completed data. MFA is then applied to each completed data leading to m different component configurations. Finally, the m configurations are combined using the STATIS method to yield one consensus solution.

Value

MIMFA returns a list that contains the following components:

compromise	the compromise configuration, a data frame with the individuals coordinates as returned by `statis` function.
configurations	a list containing the individuals coordinates as returned by `MFA` function for each imputated dataset.
strata	the original `strata` data frame containing the individual names and the corresponding stratum.
missing.ind	a list containing the name of the missing individuals.
M.total	the total number of possible imputations.

Code

The function is currently defined as:

```
MIMFA <- function(datasets, strata, ncomp = 2, M = NULL) {

  #-- checking general input parameters -----------------------------------------#
  #------------------------------------------------------------------------------#

  #-- datasets
  if (!is.list(datasets)) {
    stop("'datasets' must be a list containing at least two data tables.", call. = FALSE)
  }

  for (i in 1:length(datasets)) {
    if (length(dim(datasets[[i]])) != 2) {
      stop("datasets[[", i, "]] must be a matrix or data frame.", call. = FALSE)
    }

    if (!is.numeric(as.matrix(datasets[[i]]))) {
      stop("datasets[[", i, "]] must be a matrix or data frame.", call. = FALSE)
    }
  }

  #-- strata
    if (length(dim(strata)) != 2) {
      stop('"strata" must be a matrix or data frame with two columns.', call. = FALSE)
    }

    if (ncol(strata) != 2) {
```

```
    stop('"strata" must be with two columns.', call. = FALSE)
  }

#-- equal number of rows in all tables and strata
nb.rows <- sapply(datasets, nrow)

if (any(nb.rows != nrow(datasets[[1]]))) {
  stop("unequal number of rows in datasets.", call. = FALSE)
}

if (nb.rows[1] != nrow(strata)) {
  stop("unequal number of rows between datasets and strata.", call. = FALSE)
}

datasets <- lapply(datasets, function(x, ind) {rownames(x) <- ind; return(x)},
                   strata[, 1])
names(datasets) <- paste0("data", seq(length(datasets)))

strata <- factor(strata[, 2])
str.levels <- levels(strata)

#-- ncomp
if (is.null(ncomp) || !is.numeric(ncomp) || (ncomp < 2) || !is.finite(ncomp)) {
  stop("invalid number of components, 'ncomp'.", call. = FALSE)
}

ncomp <- round(ncomp)

#-- M (number of imputations)
if (is.null(M) || !is.numeric(M) || M < 1 || !is.finite(M)) {
  stop("invalid number of imputations, 'M'.", call. = FALSE)
}

select.comb <- function(args, nr) {
##################################################################################
## Searchs the elements of a combination from all combinations
## of the supplied vectors or factors as created by 'expand.grid'
## without creating the combinations
##
## Arguments:
##      args   list containing the vectors or factors
##        nr   the number corresponding to a combination
##################################################################################
  nargs <- length(args)
  nx <- vapply(args, length, 1)
  rep.fac <- c(1, cumprod(nx)[-nargs])

  iArgs <- seq_len(nargs)
  id <- ceiling(nr/rep.fac[iArgs]) %% nx[iArgs]
  id0 <- (id == 0)
  id[id0] <- nx[id0]

  comb <- NULL
  for (i in iArgs) {
    comb <- c(comb, args[[i]][id[i]])
  }
  comb
} ## end select.comb function

#-- creation of posible imputations in each stratum of each data ---------------#
#-------------------------------------------------------------------------------#
perm <- miss.row <- list()
k <- 1
id.data <- NULL

for (j in seq_along(datasets)) {

  if (any(is.na(datasets[[j]]))) {
    perm[[k]] <- miss.row[[k]] <- list()
    id.data <- c(id.data, names(datasets)[j])
    i <- 1

    for (s in seq_along(str.levels)) {
```

```r
      id.stratum <- (strata == str.levels[s])
      tmp <- apply(is.na(datasets[[j]][strata == str.levels[s], ]), 1, all)

      if (any(tmp)) {
        donors <- setdiff(names(tmp), names(tmp)[tmp])
        tmp2 <- t(permutations(length(donors), sum(tmp), donors))
        perm[[k]][[i]] <- tmp2 ## permutations per stratum
        miss.row[[k]][[i]]<- names(tmp)[tmp] ## missing rows
        i <- i + 1
      }
    }
    k <- k + 1
  }
}

#-- number of posible imputations
tmp <- lapply(perm, function(x) vapply(x, ncol, 1))
nb.miss.str <- unlist(lapply(tmp, length))
id.data.miss <- list()
from <- 1

for (i in seq_along(nb.miss.str)) {
  to <- sum(nb.miss.str[1:i])
  id.data.miss[[i]] <- seq(from, to)
  from <- to + 1
}

M.total <- prod(unlist(tmp))
seq.perm.data <- alply(matrix(unlist(tmp)), 1, seq)

if (is.null(M)) M <- 30
M <- min(M, M.total)

#-- selection of the donor indexes --------------------------------------------#
#------------------------------------------------------------------------------#
M.idx <- sample.int(min(M.total, 1e15), M)
id.donor <- NULL

for (i in seq_along(M.idx)) {
  id.donor <- rbind(id.donor, select.comb(seq.perm.data, M.idx[i]))
}

#-- realisation of the MFA on the imputated data ------------------------------#
#------------------------------------------------------------------------------#
variates.MFA <- list()

for (i in seq(M)) { ## nb. of imputations M
  input.data <- datasets

  for (j in seq_along(id.data)) { ## nb. of datasets
    k <- 1
    for (s in id.data.miss[[j]]) { ## nb. of strata with missing rows
      input.ind <- perm[[j]][[k]][, id.donor[i, s]]

      #-- create imputate data
      input.data[[id.data[j]]][miss.row[[j]][[k]], ] <- datasets[[id.data[j]]][input.ind, ]
      k <- k + 1
    }
  }

  #-- realisation of the MFA
  input.data <- bind_cols(as.data.frame(input.data))
  result <- MFA(input.data, group = unlist(lapply(datasets, ncol)), ncp = ncomp,
                graph = FALSE)
  variates.MFA[[i]] <- data.frame(result$ind$coord) ## MFA global coord = MFA variates
}

#-- calculation of the compromise space (STATIS method) -----------------------#
#------------------------------------------------------------------------------#
ktab.MFA <- ktab.list.df(variates.MFA)
statis.MFA <- statis(ktab.MFA, scannf = FALSE, nf = ncomp)$C.li
colnames(statis.MFA) <- colnames(variates.MFA[[1]])
```

```
  #-- results ------------------------------------------------------------------#
  #-----------------------------------------------------------------------------#
  names(miss.row) <- id.data
  strata <- data.frame('sample' = rownames(datasets[[1]]), 'stratum' = strata)
  res <- list(compromise = statis.MFA, configurations = variates.MFA,
              strata = strata, missing.ind = miss.row, M.total = M.total)
  class(res) <- "MIMFA"
  return(invisible(res))

}
```

plot.MIMFA function

plot.MIMFA	*Plot of Individuals (Experimental Units)*

Description

This function provides scatter plots for individuals (experimental units) representation from MI-MFA results.

Usage

```
plot.MIMFA(object, comp = 1:2, col.per.stratum = NULL, col.missing.ind = "white",
           plot.conf.areas = c('none', 'ellipse', 'convex.hull'),
           conf.level = 0.95, legend.title = "Stratum")
```

Arguments

`object`	object returned by `MIMFA` function.
`comp`	integer vector of length two. The components that will be used on the horizontal and the vertical axis respectively to project the individuals.
`col.per.stratum`	character vector of the same length than the number of strata, containing the color names to be used to annotate the individuals per stratum.
`plot.conf.areas`	character string indicating whether to plot `'none'`, `'ellipse'` or `'convex.hull'` confidence areas.
`conf.level`	numerical value indicating the confidence level of ellipses being plotted when `plot.conf.areas = 'ellipse'`. The default is set to 0.95, for a 95% region.
`col.missing.ind`	the fill color for imputated individuals.
`legend.title`	character. The legend title.

Details

`plot.MIMFA` function makes scatter plot for individuals representation from MI-MFA results. Each point corresponds to an individual. The individuals are colored with rapport to their stratum.

Value

An object of class `ggplot`.

Code

The function is currently defined as:

```
plot.MIMFA <- function(object,
                       comp = 1:2,
                       col.per.stratum = NULL,
                       col.missing.ind = "white",
                       plot.conf.areas = c('none', 'ellipse', 'convex.hull'),
                       conf.level = 0.95,
                       legend.title = "Stratum") {

  #-- checking general input arguments ------------------------------------------#
  #------------------------------------------------------------------------------#

  #-- comp
  ncomp <- ncol(object$compromise)

  if (length(comp) != 2) {
    stop("the length of 'comp' must be equal to 2.", call. = FALSE)
  }
  else {
    if (any(!is.finite(comp)))
      stop("invalid vector for 'comp'.", call. = FALSE)

    if (!is.numeric(comp) || any(comp < 1))
      stop("invalid vector for 'comp'.", call. = FALSE)

    if (any(comp > ncomp))
      stop("the elements of 'comp' must be smaller or equal than ", ncomp, ".",
           call. = FALSE)
  }

  comp <- round(comp)
```

```
#-- internal function for character color checking --#
#----------------------------------------------------#
isColor <- function(x) { sapply(x, function(x) {
  tryCatch(is.matrix(col2rgb(x)), error = function(e) FALSE) })
}
#----------------------------------------------------#

#-- col.per.stratum
if (is.null(col.per.stratum)) {
  col.per.stratum <- rainbow(length(levels(object$strata$stratum)))
  names(col.per.stratum) <- levels(object$strata$stratum)
}
else {
  if (length(col.per.stratum) != length(levels(object$strata$stratum))) {
    stop("'col.per.stratum' must be a color names vector of length ",
         length(levels(object$strata$stratum)), ".", call. = FALSE)
  }
  else {
    if (any(!isColor(col.per.stratum))) {
      stop("'col.per.stratum' must be a character vector of recognized colors.",
           call. = FALSE)
    }
  }
}

if (is.null(names(col.per.stratum))) {
    names(col.per.stratum) <- levels(object$strata$stratum)
}
else {
  if (any(!(names(col.per.stratum) %in% levels(object$strata$stratum))))
    stop("names of 'col.per.stratum' must be a character from: ",
         toString(levels(object$strata$stratum)),
         call. = FALSE)
}

#-- col.missing.ind
if (length(as.vector(col.missing.ind)) != 1) {
  stop("'col.missing.ind' must be a character of recognized colors.",
       call. = FALSE)
}

if (!isColor(col.missing.ind) | is.na(col.missing.ind)) {
  stop("'col.missing.ind' must be a character of recognized colors.",
       call. = FALSE)
}

#-- plot.conf.areas
plot.conf.areas <- match.arg(plot.conf.areas)

#-- legend.title
legend.title <- as.graphicsAnnot(legend.title)


#-- individuals scatter plot ---------------------------------------------------#
#-------------------------------------------------------------------------------#
compromise <- object$compromise
n <- nrow(compromise)
miss <- rep("not", n)
miss[object$strata$sample %in% unlist(object$missing.ind)] <- "yes"
stratum <- factor(object$strata$stratum, levels = names(col.per.stratum))

if (plot.conf.areas == 'none') {
  df <- data.frame(x = compromise[, comp[1]], y = compromise[, comp[2]],
                   ind = object$strata$sample, stratum = stratum,
                   missing = miss)

  df$ind.miss <- paste(df$ind, df$miss, sep = ".")
  df$ind.miss[df$miss == "not"] <- as.character(df$stratum[df$miss == "not"])
  df$ind.miss <- as.factor(df$ind.miss)

  ind.cols <- col.per.stratum[df$stratum]
```

```
  g <- ggplot(data = df, aes(x, y, fill = stratum, color = stratum)) +
    theme_bw() +
    geom_hline(yintercept = 0, color = 'grey30', size = 0.5, linetype = 2) +
    geom_vline(xintercept = 0, color = 'grey30', size = 0.5, linetype = 2) +
    geom_point(data = df[df$missing == "not", ], size = 3, shape = 21) +
    geom_point(data = df[df$missing == "yes", ], size = 3, shape = 21,
               fill = col.missing.ind) +
    scale_colour_manual(values = ind.cols) +
    scale_fill_manual(name = legend.title, values = ind.cols) +
    guides(colour = "none") +
    labs(x = paste0('Dim ', comp[1]), y = paste0('Dim ', comp[2]))
}

#-- confidence ellipses --------------------------------------------------------#
#------------------------------------------------------------------------------#
if (plot.conf.areas == 'ellipse') {
  df <- data.frame(x = compromise[, comp[1]], y = compromise[, comp[2]],
                   ind = object$strata$sample, stratum = stratum,
                   missing = miss, config = rep("compromise", n))

  m <- length(object$configuration)

  for (j in 1:m) {
    X <- as.matrix(object$configuration[[j]])
    P <- X %*% solve(t(X) %*% X) %*% t(X)
    traj <- P %*% as.matrix(compromise)

    temp <- data.frame(x = traj[, comp[1]], y = traj[, comp[2]],
                       ind = object$strata$sample, stratum = stratum,
                       missing = miss, config = rep("imputed", n))
    df <- rbind(df, temp)
  }

  df$ind.conf <- paste(df$ind, df$config, sep = ".")
  df$ind.conf[df$config == "compromise"] <-
    as.character(df$stratum[df$config == "compromise"])
  df$ind.conf <- as.factor(df$ind.conf)

  ind.cols <- col.per.stratum[df$stratum]
  names(ind.cols) <- df$ind.conf

  g <- ggplot(data = df, aes(x, y, colour = ind.conf, fill = ind.conf)) +
    theme_bw() +
    geom_hline(yintercept = 0, color = 'grey30', size = 0.5, linetype = 2) +
    geom_vline(xintercept = 0, color = 'grey30', size = 0.5, linetype = 2) +
    stat_ellipse(data = df[df$conf == "imputed", ], level = conf.level,
                 geom = 'polygon', alpha = 0.1) +
    geom_point(data = df[df$missing == "not" & df$conf == "compromise", ],
               size = 3, shape = 21) +
    geom_point(data = df[df$missing == "yes" & df$conf == "compromise", ],
               size = 3, shape = 21, fill = col.missing.ind, show.legend = FALSE) +
    scale_colour_manual(breaks = df$ind.cong[df$conf == "compromise"], values = ind.cols) +
    scale_fill_manual(name = legend.title, breaks = df$ind.conf[df$conf == "compromise"],
                      values = ind.cols) +
    labs(x = paste0('Dim ', comp[1]), y = paste0('Dim ', comp[2]))
}

#-- convex hulls ---------------------------------------------------------------#
#------------------------------------------------------------------------------#
if (plot.conf.areas == 'convex.hull') {
  df <- data.frame(x = compromise[, comp[1]], y = compromise[, comp[2]],
                   ind = object$strata$sample, stratum = stratum,
                   missing = miss, config = rep("compromise", n))

  m <- length(object$configuration)

  for (j in 1:m) {
    X <- as.matrix(object$configuration[[j]])
    P <- X %*% solve(t(X) %*% X) %*% t(X)
    traj <- P %*% as.matrix(compromise)

    temp <- data.frame(x = traj[, comp[1]], y = traj[, comp[2]],
                       ind = object$strata$sample, stratum = stratum,
```

```
                        missing = miss, config = rep("imputed", n))
      df <- rbind(df, temp)
    }

    df$ind.conf <- paste(df$ind, df$config, sep = ".")
    df$ind.conf[df$config == "compromise"] <-
      as.character(df$stratum[df$config == "compromise"])
    df$ind.conf <- as.factor(df$ind.conf)

    find_hull <- function(df) df[chull(df$x, df$y), ]
    hulls <- ddply(df, "ind", find_hull)

    ind.cols <- col.per.stratum[df$stratum]
    names(ind.cols) <- df$ind.conf

    g <- ggplot(data = df, aes(x, y, colour = ind.conf, fill = ind.conf)) +
      theme_bw() +
      geom_hline(yintercept = 0, color = 'grey30', size = 0.5, linetype = 2) +
      geom_vline(xintercept = 0, color = 'grey30', size = 0.5, linetype = 2) +
      geom_polygon(data = hulls, alpha = 0.1) +
      geom_point(data = df[df$missing == "not" & df$conf == "compromise", ],
                 size = 3, shape = 21) +
      geom_point(data = df[df$missing == "yes" & df$conf == "compromise", ],
                 size = 3, shape = 21, fill = col.missing.ind, show.legend = FALSE) +
      scale_colour_manual(breaks = df$ind.cong[df$conf == "compromise"], values = ind.cols) +
      scale_fill_manual(name = legend.title, breaks = df$ind.conf[df$conf == "compromise"],
                        values = ind.cols) +
      labs(x = paste0('Dim ', comp[1]), y = paste0('Dim ', comp[2]))
  }

  print(g)
  return(invisible(g))
}
```

`tuneM` function

tuneM	*Tune the appropriate number of imputations in MIMFA*

Description

The `tuneM` function tune the appropriate number of imputations in MIMFA.

Usage

```
tuneM(datasets, strata, ncomp = 2, M.max = 30, inc = 5, N = 10, tol = 1e-06)
```

Arguments

`datasets`	a list of data tables containing the studied datasets with missing rows. Tables in the list should be arranged in samples $\times$ variables, with samples order matching in all data tables.
`strata`	data frame with two columns, the first one containing the individual names and the second containing the corresponding condition (stratum).
`ncomp`	number of MFA components. Default to 2.
`M.max`	integer, maximum number of imputations.
`inc`	integer, increment of the number of imputations.
`N`	integer, the number of replicate collections of M imputations.
`tol`	convergence criterion stopping value.

Details

The appropriate number of imputations is informally determined by carrying out MI-MFA on N replicate collections of $M_l = \texttt{inc}*l$ imputations for $l = 1, 2, \ldots,$ with $M_1 < M_2 < \cdots < M_{total}$, until the estimate compromise configurations are stabilized or $M_l = \texttt{M.max}$.

Value

An object of class `ggplot`.

Code

The function is currently defined as:

```
tuneM <- function(datasets, strata, ncomp = 2, M.max = 30, inc = 5, N = 10, tol = 1e-06) {

  #-- checking general input parameters -----------------------------------------#
  #------------------------------------------------------------------------------#

  #-- datasets
  if (!is.list(datasets)) {
    stop("'datasets' must be a list containing at least two data tables.", call. = FALSE)
  }

  for (i in 1:length(datasets)) {
    if (length(dim(datasets[[i]])) != 2) {
      stop("datasets[[", i, "]] must be a matrix or data frame.", call. = FALSE)
    }

    if (!is.numeric(as.matrix(datasets[[i]]))) {
      stop("datasets[[", i, "]] must be a matrix or data frame.", call. = FALSE)
    }
  }

  #-- strata
  if (length(dim(strata)) != 2) {
    stop('"strata" must be a matrix or data frame with two columns.', call. = FALSE)
  }

  if (ncol(strata) != 2) {
    stop('"strata" must be with two columns.', call. = FALSE)
  }

  #-- equal number of rows in all tables and strata
  nb.rows <- sapply(datasets, nrow)
```

```
if (any(nb.rows != nrow(datasets[[1]]))) {
  stop("unequal number of rows in datasets.", call. = FALSE)
}

if (nb.rows[1] != nrow(strata)) {
  stop("unequal number of rows between datasets and strata.", call. = FALSE)
}

datasets <- lapply(datasets, function(x, ind) {rownames(x) <- ind; return(x)},
                   strata[, 1])
names(datasets) <- paste0("data", seq(length(datasets)))

strata <- factor(strata[, 2])
str.levels <- levels(strata)

#-- ncomp
if (is.null(ncomp) || !is.numeric(ncomp) || (ncomp < 2) || !is.finite(ncomp)) {
  stop("invalid number of components, 'ncomp'.", call. = FALSE)
}

ncomp <- round(ncomp)

#-- M.max (number max of imputations)
  if (is.null(M.max) || !is.numeric(M.max) || M.max < 1 || !is.finite(M.max)) {
    stop("invalid maximum number of imputations, 'M.max'.", call. = FALSE)
  }

#-- increment of M
if (is.null(inc) || !is.numeric(inc) || inc < 1 || !is.finite(inc)) {
  stop("invalid increment of M, 'inc'.", call. = FALSE)
}

if (inc > round(M.max/2 + 0.5)) {
  stop("'inc' must be less than or equal to ", round(M.max/2 + 0.5), ".", call. = FALSE)
}

M <- N * M.max

select.comb <- function(args, nr) {
  ##################################################################################
  ## Searchs the elements of a combination from all combinations
  ## of the supplied vectors or factors as created by 'expand.grid'
  ## without creating the combinations
  ##
  ## Arguments:
  ##      args   list containing the vectors or factors
  ##        nr   the number corresponding to a combination
  ##################################################################################
  nargs <- length(args)
  nx <- vapply(args, length, 1)
  rep.fac <- c(1, cumprod(nx)[-nargs])

  iArgs <- seq_len(nargs)
  id <- ceiling(nr/rep.fac[iArgs]) %% nx[iArgs]
  id0 <- (id == 0)
  id[id0] <- nx[id0]

  comb <- NULL
  for (i in iArgs) {
    comb <- c(comb, args[[i]][id[i]])
  }
  comb
} ## end select.comb function

#-- creation of posible imputations in each stratum of each data ---------------#
#-------------------------------------------------------------------------------#
perm <- miss.row <- list()
k <- 1
id.data <- NULL

for (j in seq_along(datasets)) {

  if (any(is.na(datasets[[j]]))) {
```

```
      perm[[k]] <- miss.row[[k]] <- list()
      id.data <- c(id.data, names(datasets)[j])
      i <- 1

      for (s in seq_along(str.levels)) {
        id.stratum <- (strata == str.levels[s])
        tmp <- apply(is.na(datasets[[j]][strata == str.levels[s], ]), 1, all)

        if (any(tmp)) {
          donors <- setdiff(names(tmp), names(tmp)[tmp])
          tmp2 <- t(permutations(length(donors), sum(tmp), donors))
          perm[[k]][[i]] <- tmp2 ## permutations per stratum
          miss.row[[k]][[i]]<- names(tmp)[tmp] ## missing rows
          i <- i + 1
        }
      }
      k <- k + 1
    }
  }

  #-- number of posible imputations
  tmp <- lapply(perm, function(x) vapply(x, ncol, 1))
  nb.miss.str <- unlist(lapply(tmp, length))
  id.data.miss <- list()
  from <- 1

  for (i in seq_along(nb.miss.str)) {
    to <- sum(nb.miss.str[1:i])
    id.data.miss[[i]] <- seq(from, to)
    from <- to + 1
  }

  M.total <- prod(unlist(tmp))

  #-- cheking N * M.max < M.total
  if (M > M.total) {
    stop("'N * M.max' must be less than ", M.total, ".", call. = FALSE)
  }

  seq.perm.data <- alply(matrix(unlist(tmp)), 1, seq)

  #-- selection of the donor indexes --------------------------------------------#
  #------------------------------------------------------------------------------#
  M.idx <- sample.int(min(M.total, 1e15), M)
  id.donor <- NULL

  for (i in seq_along(M.idx)) {
    id.donor <- rbind(id.donor, select.comb(seq.perm.data, M.idx[i]))
  }

  #-- iterative approach --------------------------------------------------------------#
  #------------------------------------------------------------------------------------#
  variates.MFA <- list()
  Ml <- seq(inc, M.max, by = inc)
  nbMl <- length(Ml)
  ave.RV.coef <- se.RV.coef <- NULL

  #-- initial configuration M_0
  conf0 <- list()
  m <- 1
  In <- split(1:(m * inc * N), rep(1:N, length = m * inc * N))

  #-- realisation of the MFA on the imputated data
  for (i in unlist(In)) { ## nb. of imputations M
    input.data <- datasets

    for (j in seq_along(id.data)) { ## nb. of datasets
      k <- 1
      for (s in id.data.miss[[j]]) { ## nb. of strata with missing rows
        input.ind <- perm[[j]][[k]][, id.donor[i, s]]

        #-- create imputate data
        input.data[[id.data[j]]][miss.row[[j]][[k]], ] <- datasets[[id.data[j]]][input.ind, ]
```

```
      k <- k + 1
    }
  }

  #-- realisation of the MFA
  imput.data <- bind_cols(as.data.frame(imput.data))
  result <- MFA(imput.data, group = unlist(lapply(datasets, ncol)), ncp = ncomp,
                graph = FALSE)
  variates.MFA[[i]] <- data.frame(result$ind$coord) ## MFA global coord = MFA variates
}

for (n in 1:N) {
  #-- calculation of the compromise space (STATIS method)
  ktab.MFA <- ktab.list.df(variates.MFA[In[[n]]])
  conf0[[n]] <- statis(ktab.MFA, scannf = FALSE, nf = ncomp)$C.li
}

#-- configurations for M_l, l > 1
old.ave <- -1
m <- 2
repeat {
  In <- split(1:(m * inc * N), rep(1:N, length = m * inc * N))
  subIn <- split((((m - 1) * inc * N) + 1):(m * inc * N), rep(1:N, length = inc * N))
  RV.coef <- NULL

  #-- realisation of the MFA on the imputated data
  for (i in unlist(subIn)) { ## nb. of imputations M
    imput.data <- datasets

    for (j in seq_along(id.data)) { ## nb. of datasets
      k <- 1
      for (s in id.data.miss[[j]]) { ## nb. of strata with missing rows
        imput.ind <- perm[[j]][[k]][, id.donor[i, s]]

        #-- create imputate data
        imput.data[[id.data[j]]][miss.row[[j]][[k]], ] <- datasets[[id.data[j]]][imput.ind, ]
        k <- k + 1
      }
    }

    #-- realisation of the MFA
    imput.data <- bind_cols(as.data.frame(imput.data))
    result <- MFA(imput.data, group = unlist(lapply(datasets, ncol)), ncp = ncomp,
                  graph = FALSE)
    variates.MFA[[i]] <- data.frame(result$ind$coord) ## MFA global coord = MFA variates
  }

  for (n in 1:N) {
    #-- calculation of the compromise space (STATIS method)
    ktab.MFA <- ktab.list.df(variates.MFA[In[[n]]])
    conf <- statis(ktab.MFA, scannf = FALSE, nf = ncomp)$C.li
    RV.coef <- c(RV.coef, coeffRV(conf0[[n]], conf)$rv)
    conf0[[n]] <- conf
  }

  ave.RV.coef <- c(ave.RV.coef, mean(RV.coef))
  se.RV.coef <- c(se.RV.coef, sqrt(var(RV.coef)))

  if (m >= nbMl | abs(old.ave - ave.RV.coef[m - 1]) < tol) break

  old.ave <- ave.RV.coef[m - 1]
  m <- m + 1
}

#-- graphic representation ----------------------------------------------------------#
#-----------------------------------------------------------------------------------#
df <- data.frame(x = 1:length(ave.RV.coef), ave = ave.RV.coef, se = se.RV.coef)
lab <- paste0("(", Ml[1:(nbMl - 1)], ",", Ml[2:nbMl], ")")

g <- ggplot(df, aes(x = x, y = ave)) +
  geom_point(size = 2.5) + theme_bw() +
  geom_errorbar(aes(ymax = ave + se, ymin = ave - se), width = 0.15) +
  theme(panel.margin = unit(2, "lines")) +
```

```
    labs(x = expression(paste("number of imputations (", italic(M[l]), ", ", italic(M[l + 1]), ")")),
         y = 'RV coefficient\n') +
    scale_x_continuous(breaks = seq(nbMl - 1), labels = lab) +
    theme(axis.title = element_text(size = 18)) +
    theme(axis.text.x = element_text(size = 16, angle = 45, hjust = 1, vjust = 1)) +
    theme(axis.text.y = element_text(size = 14))
  print(g)

  #-- results -------------------------------------------------------------------#
  #------------------------------------------------------------------------------#
  return(invisible(g))
}
```

2 Examples

Requried packages

```
library(FactoMineR)
library(ade4)
library(plyr)
library(dplyr)
library(gtools)
library(ggplot2)
library(mixOmics)
library(omicade4)
library(rcellminer)
library(rcellminerData)
library(RColorBrewer)
```

Example 1: partial figure 3 of the paper

```
#-- the data -----------------------------------------------------------------------#
#-----------------------------------------------------------------------------------#
#-- load data
data(liver.toxicity)

#-- treatment data
dose <- rep("low", nrow(liver.toxicity$treatment))
dose[liver.toxicity$treatment$Dose.Group > 1000] = "high"
condition <- paste(dose, liver.toxicity$treatment$Time.Group, sep = "-")
treatment <- data.frame(id = rownames(liver.toxicity$treatment),
                        condition = condition)

#-- trascriptomic data
trans <- liver.toxicity$gene
rownames(trans) <- rownames(liver.toxicity$treatment)

#-- clinical data
clinic <- liver.toxicity$clinic

#-- incomplete trascriptomic data
trans.incompl <- trans
trans.incompl[c(1, 7, 9, 10, 13, 15, 16, 19, 20, 25, 28, 31, 35,
                36, 39, 40, 45, 46, 48, 49, 57, 58, 60, 62), ] <- NA

#-- liste containing both data sets
datasets <- list(trans.incompl, clinic)

#-- performing MIMFA ----------------------------------------------------------------#
#-----------------------------------------------------------------------------------#
res <- MIMFA(datasets, treatment, ncomp = 2, M = 30)

#-- plot the results ----------------------------------------------------------------#
#-----------------------------------------------------------------------------------#
#-- colors for treatments
cols <- brewer.pal(11,"RdYlGn")[c(8:11, 4:1)]
names(cols) <- c("low-6", "low-18", "low-24", "low-48",
                 "high-6", "high-18", "high-24", "high-48")
```

```
plot.MIMFA(res, comp = 1:2, col.per.stratum = cols,
           col.missing.ind = "white",
           plot.conf.areas = 'none',
           legend.title = "Treatment")
```

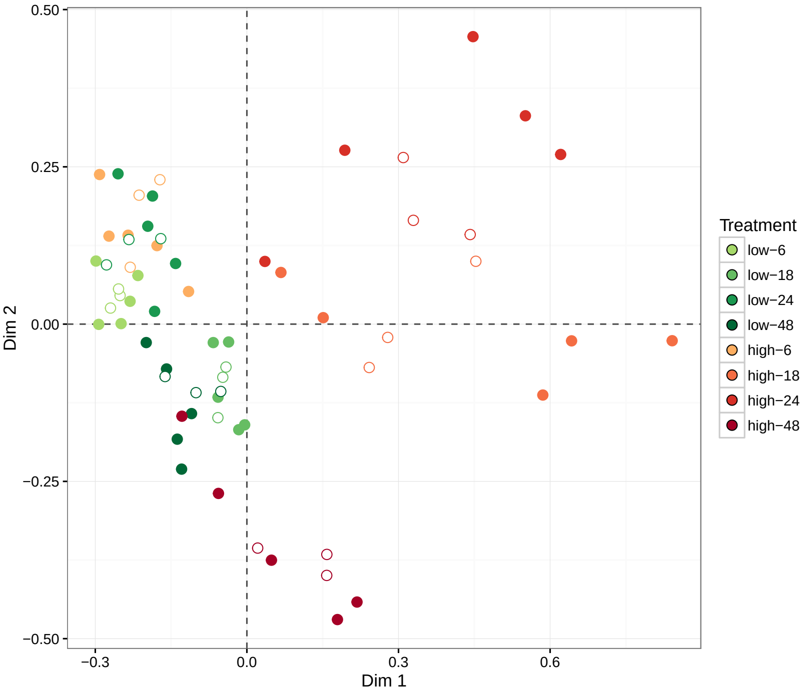

Example 2: How many imputations? (small example)

The implementation of this example can take some minutes.

```
#-- incomplete trascriptomic data
trans.incompl <- trans
trans.incompl[c(1, 3, 10), ] <- NA

#-- liste containing both data sets
datasets <- list(trans.incompl, clinic)

#-- tuning M
tuneM(datasets, treatment, ncomp = 2, M.max = 36, inc = 4, N = 5, tol = 1e-09)
```

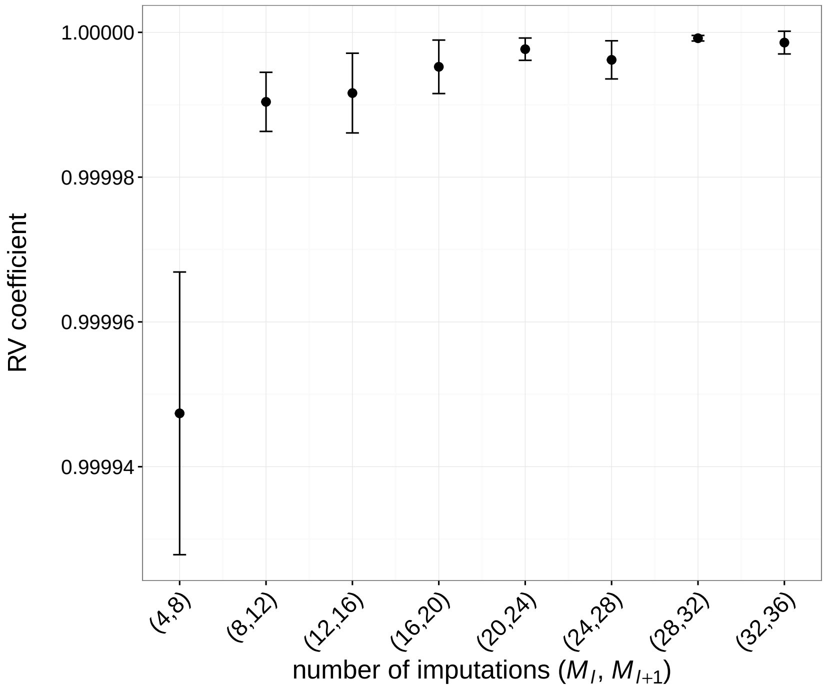

Example 3: partial figure 7 of the paper

```
#-- the data ----------------------------------------------------------------------#
#---------------------------------------------------------------------------------#
#-- load data
data(NCI60_4arrays)
data(molData)

#-- a subset of microarray gene expression of the NCI 60 cell lines
trans <- t(NCI60_4arrays$agilent)

#-- a subset of proteomic data of the NCI 60 cell lines
prote <- t(exprs(molData@eSetList$pro))

#-- cell line information: the strata dataframe
sample <- getSampleData(molData)$Name
strata <- data.frame(sample = sample, class = sample, stringsAsFactors = FALSE)
for (i in 1:length(sample)) {
  strata$class[i] <- strsplit(sample[i], "[:]")[[1]][1]
  rownames(trans)[i] <- paste(strsplit(rownames(trans)[i], "[.]")[[1]], collapse = ":")
}
strata$class <- as.factor(strata$class)

#-- check whether samples are ordered correctly
identical(rownames(trans), rownames(prote))

## [1] TRUE

#-- incomplete trascriptomic data
trans.incompl <- trans
trans.incompl[c(6, 8, 14, 15, 18, 23:28, 35, 37, 44, 45, 53, 55, 60), ] <- NA

#-- liste containing both data sets
datasets <- list(trans.incompl, prote)

#-- performing MIMFA ---------------------------------------------------------------#
#---------------------------------------------------------------------------------#
res <- MIMFA(datasets, strata, ncomp = 2, M = 30)

#-- plot the results ---------------------------------------------------------------#
#---------------------------------------------------------------------------------#
#-- colors for cancer type
CO  <- rgb(236, 40, 45, maxColorValue = 255)
CNS <- rgb(135, 144, 77, maxColorValue = 255)
LE  <- rgb(47, 188, 197, maxColorValue = 255)
ME  <- rgb(128, 62, 16, maxColorValue = 255)
RE  <- rgb(0, 0, 0, maxColorValue = 255)
OV  <- rgb(238, 154, 54, maxColorValue = 255)
PR  <- rgb(173, 19, 156, maxColorValue = 255)
LC  <- rgb(124, 124, 124, maxColorValue = 255)
BR  <- rgb(31, 77, 161, maxColorValue = 255)

cols <- c("BR" = BR, "CNS" = CNS, "CO" = CO, "LC" = LC, "LE" = LE,
          "ME" = ME, "OV" = OV, "PR" = PR, "RE" = RE)
```

```
plot.MIMFA(res, comp = 1:2, col.per.stratum = cols,
           col.missing.ind = "white",
           plot.conf.areas = 'none',
           legend.title = "Cancer type")
```

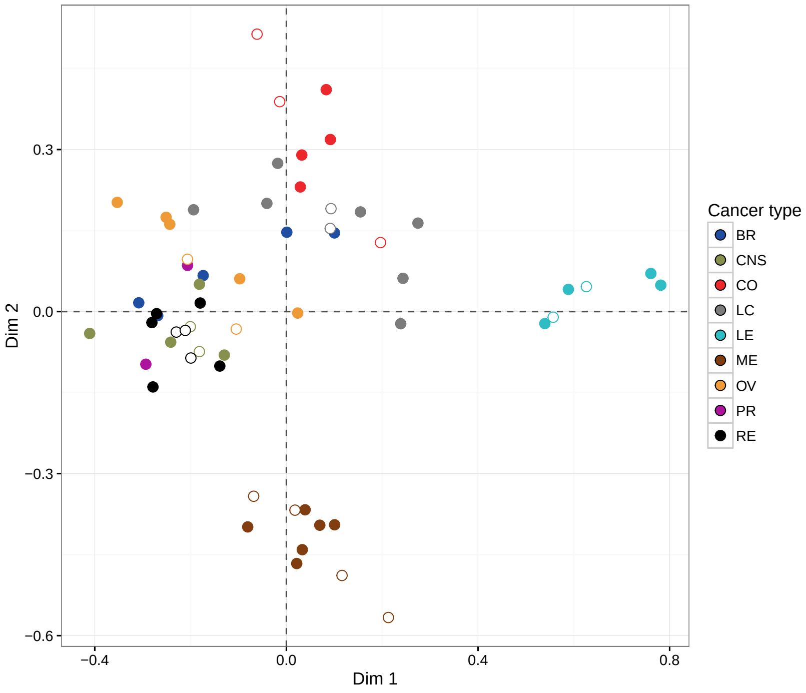


```
#-- confidence ellipses
plot.MIMFA(res, comp = 1:2, col.per.stratum = cols,
           col.missing.ind = "white",
           plot.conf.areas = 'ellipse',
           legend.title = "Cancer type")
```

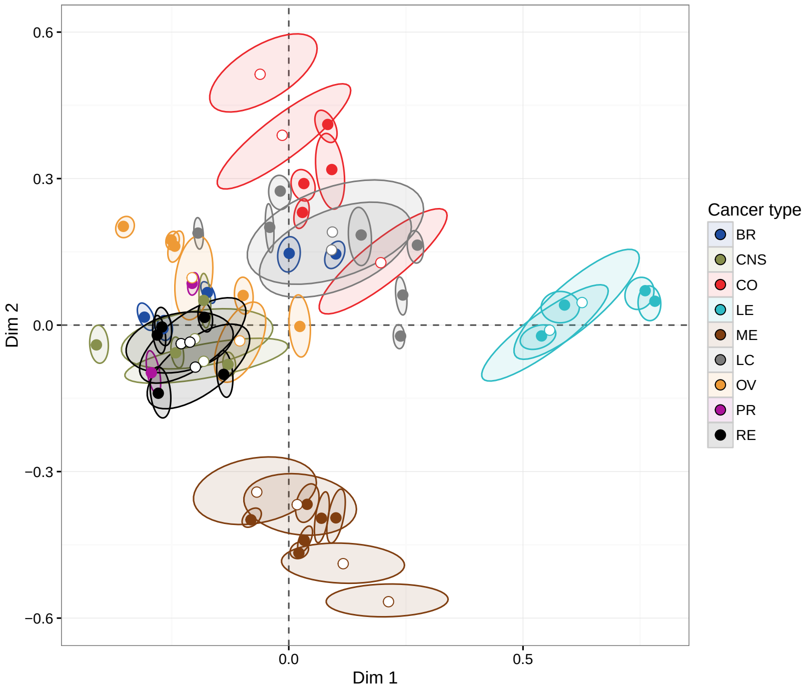

```
#-- convex hulls
plot.MIMFA(res, comp = 1:2, col.per.stratum = cols,
           col.missing.ind = "white",
           plot.conf.areas = 'convex.hull',
           legend.title = "Cancer type")
```

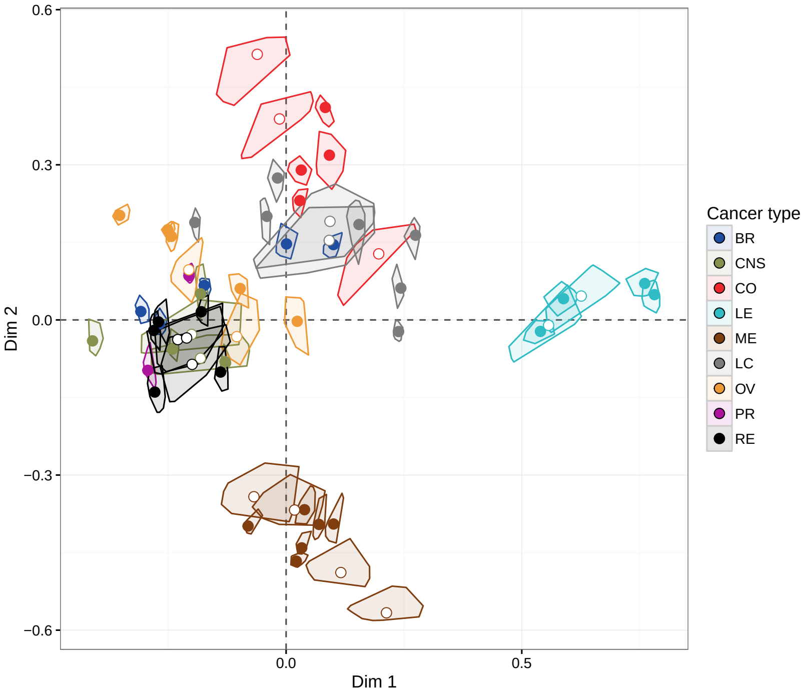

3 Session information

The following is the session info that generated this document:

```
R version 3.2.0 (2015-04-16)
Platform: x86_64-pc-linux-gnu (64-bit)
Running under: Ubuntu precise (12.04.5 LTS)

locale:
 [1] LC_CTYPE=fr_FR.UTF-8       LC_NUMERIC=C               LC_TIME=fr_FR.UTF-8
 [4] LC_COLLATE=fr_FR.UTF-8     LC_MONETARY=fr_FR.UTF-8    LC_MESSAGES=fr_FR.UTF-8
 [7] LC_PAPER=fr_FR.UTF-8       LC_NAME=C                  LC_ADDRESS=C
[10] LC_TELEPHONE=C             LC_MEASUREMENT=fr_FR.UTF-8 LC_IDENTIFICATION=C

attached base packages:
[1] parallel  stats     graphics  grDevices utils     datasets  methods   base

other attached packages:
 [1] RColorBrewer_1.1-2   rcellminer_1.2.3     rcellminerData_1.2.2 rcdk_3.3.2
 [5] fingerprint_3.5.2    Biobase_2.30.0       BiocGenerics_0.16.1  omicade4_1.10.0
 [9] mixOmics_6.0.0       lattice_0.20-33      MASS_7.3-45          ggplot2_2.1.0
[13] gtools_3.5.0         dplyr_0.5.0          plyr_1.8.4           ade4_1.7-4
[17] FactoMineR_1.33      printr_0.0.5         knitr_1.13

loaded via a namespace (and not attached):
 [1] made4_1.44.0         reshape2_1.4.1       rJava_0.9-8          colorspace_1.2-6
 [5] htmltools_0.3.5      chron_2.3-47         DBI_0.4-1            stringr_1.0.0
 [9] munsell_0.4.3        gtable_0.2.0         rcdklibs_1.5.13      caTools_1.17.1
[13] leaps_2.9            evaluate_0.9         labeling_0.3         httpuv_1.3.3
[17] highr_0.6            Rcpp_0.12.5          xtable_1.8-2         KernSmooth_2.23-15
[21] corpcor_1.6.8        scales_0.4.0         flashClust_1.01-2    formatR_1.4
[25] gdata_2.17.0         scatterplot3d_0.3-37 mime_0.4             gplots_3.0.1
[29] ellipse_0.3-8        BiocStyle_1.8.0      png_0.1-7            digest_0.6.9
[33] stringi_1.1.1        shiny_0.13.2         grid_3.2.0           tools_3.2.0
[37] bitops_1.0-6         magrittr_1.5         rgl_0.95.1441        tibble_1.0
[41] cluster_2.0.4        tidyr_0.5.1          data.table_1.9.6     assertthat_0.1
[45] iterators_1.0.8      R6_2.1.2             igraph_1.0.1
```
